# Preparation of Novel Pyrazolo[4,3-*e*]tetrazolo[1,5-*b*][1,2,4]triazine Sulfonamides and Their Experimental and Computational Biological Studies

**DOI:** 10.3390/ijms23115892

**Published:** 2022-05-24

**Authors:** Mateusz Kciuk, Somdutt Mujwar, Anna Szymanowska, Beata Marciniak, Karol Bukowski, Mariusz Mojzych, Renata Kontek

**Affiliations:** 1 Department of Molecular Biotechnology and Genetics, University of Lodz, 90-237 Lodz, Poland; beata.marciniak@biol.uni.lodz.pl (B.M.); karol.bukowski@edu.uni.lodz.pl (K.B.); renata.kontek@biol.uni.lodz.pl (R.K.); 2 Doctoral School of Exact and Natural Sciences, University of Lodz, 90-237 Lodz, Poland; 3 M.M. College of Pharmacy, Maharishi Markandeshwar (Deemed to be University) Mullana, Ambala 133207, India; somduttmujwar@gmail.com; 4 Department of Chemistry, Siedlce University of Natural Sciences and Humanities, 08-110 Siedlce, Poland; anna.szymanowska@umb.edu.pl (A.S.); mariusz.mojzych@uph.edu.pl (M.M.)

**Keywords:** cancer cells, cytotoxicity, apoptosis, pyrazolo[4,3-*e*]tetrazolo[1,5-*b*][1,2,4]triazine, sulfonamides

## Abstract

Pyrazolo[4,3-*e*]tetrazolo[1,5-*b*][1,2,4]triazine sulfonamides constitute a novel class of heterocyclic compounds with broad biological activity, including anticancer properties. Investigated in this study, MM-compounds (**MM134**, **MM136**, **MM137**, and **MM139**) exhibited cytotoxic and proapoptotic activity against cancer cell lines (BxPC-3, PC-3, and HCT-116) in nanomolar concentrations without causing cytotoxicity in normal cells (L929 and WI38). In silico predictions indicate that tested compounds exhibit favorable pharmacokinetic profiles and may exert anticancer activity through the inhibition of BTK kinase, the AKT-mTOR pathway and PD1-PD-L1 interaction. Our findings point out that these sulfonamide derivatives may constitute a source of new anticancer drugs after optimization.

## 1. Introduction

The chemistry of heterocyclic compounds is still a developing field that offers many opportunities for novel drug discovery, which is confirmed by the fact that many modern drugs used in medicine contain a heterocyclic core. Moreover, new methods of synthesis and modern organic chemistry techniques have led to the rapid expansion of this branch of science. Anticancer drug development is one of the main objectives of heterocyclic chemistry [1].

One of the common strategies used in anticancer drug development is the synthesis of antimetabolites that structurally resemble naturally occurring key substrates of metabolic processes. Blocking of these crucial processes slows down the proliferation of cells and triggers their apoptotic cell death [2,3,4]. Apoptosis is considered to be a crucial component of the vital processes of living organisms. It is responsible for the proper development and functioning of the immune system. Apoptosis deregulation results in the development of a multitude of human conditions, including neurodegenerative diseases, autoimmune disorders, and many types of cancer. Apoptosis is a genetically designed process of active cell destruction. Cells undergoing apoptosis remain intact without lysis, inflammation, and causing damage to neighboring cells. The activation of apoptosis pathways is a key mechanism of action for many cytotoxic drugs. Moreover, defects in apoptosis signaling contribute to drug resistance in many tumors. Cytotoxic drugs activate either the mitochondrial (intrinsic) or death receptor (extrinsic) apoptosis pathway [5,6]. Nowadays, most anticancer drugs exhibit proapoptotic properties. Moreover, the majority of them are designed to take advantage of cell division in order to achieve selective action. The selectivity is based on the more rapid division of cancer cells compared to their normal counterparts. However, these molecules are not sufficiently selective for cancer cells and result in toxicity to normal cells, which provokes serious consequences for patients [6]. Therefore, there is an urgent need to explore both the cytotoxic and proapoptotic properties of new potential anticancer drugs. One of the characteristic changes occurring during apoptosis is the translocation of phosphatidylserine from the inner side of a cell membrane to the surface of a cell. This allows rapid and accurate estimation of proapoptotic properties of anticancer compounds with the use of annexin V, which forms conjugates with a fluorescent dye (FITC) and allows the detection of phosphatidylserine exposition on the cell surface with the use of flow cytometry [7,8].

Pyrazolotriazines were identified as biologically active compounds with inhibitory activity towards histone deacetylases [9], metalloproteinases [10], tubulin [11], urease and tyrosinase [12,13]. Moreover, some of them inhibit protein kinases engaged in pivotal signaling pathways driving cancer cell proliferation, including ABL kinase [14,15], cyclin-dependent kinases (CDKs) [16,17], casein kinase 2 (CK2) [18,19], and glycogen synthase kinase 3 (GSK3) [20]. The addition of the sulfonamide moiety to the pyrazolotriazine scaffold allowed for the broadening of the array of possible molecular targets of these compounds [21]. For example, pyrazolo[4,3-*e*][1,2,4]triazine derivatives have been shown to have ABL protein kinase inhibitory activity in the micromolar concentration range (IC_50_ = 5.8–5.9 µM). Furthermore, sulfonamide-bearing compounds are the most abundant class of carbonic anhydrase (CA) inhibitors. The inhibition of tumor isoforms of CA (CA-IX and XII) has been shown to significantly reduce the survival of hypoxic tumors. Nowadays, sulfonamide compounds have been tested in clinical trials, with SLC-0011 being one of the most prominent examples of this type of agents [22]. The best results against hCA-IX were observed with pyrazolo[4,3-*e*][1,2,4]triazines with dissociation constants (KI values) of 23.7 and 26.5 nM, which were similar to reference sulfonamide compound acetazolamide (KI = 25 nM). In contrast, all derivatives tested in these studies showed a good inhibition of CA-XII with KIs in the range of 5.3 nM to 9.0 nM [23,24].

The findings of experimental research published to date have demonstrated that a variety of derivatives of the pyrazolo-triazine heterocyclic system exhibit a wide range of biological activity, including anticancer activity. Tricyclic pyrazolo[4,3-*e*][1,2,4] triazines fused with a triazole or tetrazole ring are of special interest. They represent novel heterocyclic systems with strong anticancer activity and suggest that they may be a source of new chemotherapeutic drugs in the future. The biological activity of a number of the pyrazolo[4,3-*e*][1,2,4]triazines described in the literature was recently reviewed by the Mojzych group [16].

More recently, a novel **MM129** (pyrazolo[4,3-*e*]tetrazolo[1,5-*b*][1,2,4]triazine sulfonamide) has been shown to efficiently suppress cell viability through the inhibition of Bruton’s tyrosine kinase (BTK). Activation of antiapoptotic pathways has been shown to play a significant role in the growth of tumors, and BTK has been implicated in this process. In turn, BTK inhibition can trigger apoptosis activation in a variety of cell types. Compared to the routinely used chemotherapeutic agent (5-fluorouracil, 5-FU), **MM129** exhibited a substantially higher anticancer efficacy at a relatively low dose. Apoptosis was found to be the primary response of colorectal cancer cells (DLD-1 and HT-29 cell lines) to **MM129** treatment. **MM129** also inhibited tumor development in a zebrafish embryo xenograft model, where it had a notably synergistic anticancer impact when given in conjunction with 5-FU [25].

It was found that **MM129** possesses antitumor activity in xenograft mouse models of colon cancer. The mechanistic analysis found that **MM129** not only has the potential to inhibit intracellular pathways that promote carcinogenesis but also has the potential to decrease the protein levels of PD-L1 [26]. Immune checkpoint pathways, such as the programmed death receptor-1 and programmed death ligand-1 (PD-1/PD-L1) signaling pathway, are critical in regulating self-tolerance and controlling self-damage, and can be manipulated by cancer cells to avoid immune surveillance [27,28]. Recent clinical trials have demonstrated the efficacy of PD-1/PD-L1-targeted therapy in a variety of malignancies, heralding the beginning of a new era in cancer immunotherapy [29]. Furthermore, the treatment of cells with this novel derivative in combination with 5-FU appears to sensitize tumor cells to this extensively used chemotherapeutic agent. In both DLD-1 and HT-29 cells, exposure to **MM129** resulted in a decrease in the expression of AKT and mTOR serine/threonine-protein kinases [26]. The AKT/mTOR pathway plays a crucial role in the regulation of a variety of processes associated with cell growth, metabolism, survival, and proliferation. Increased expression of the mTOR kinase is observed in many types of cancer. The activation of the mTOR pathway is responsible for the stimulation of tumor growth and metastasis. A total of 60–70% of human colon malignancies have been found to have over-activated AKT-mTOR signaling [30,31,32]. Many mTOR inhibitors have been approved by the FDA for the treatment of cancer, but a large number of them are still being investigated in numerous clinical trials [33,34]. Both **MM-129** and 5-FU treatment resulted in a significant upregulation of cellular tumor antigen p53 (TP53), as well as a corresponding downregulation of CDK2, in both cell lines after 24 h exposure to the compounds. This alteration was most likely the cause of the cell cycle arrest [26].

**MM129** possesses good pharmacokinetic properties, with rapid absorption and bioavailability of 68.6% after intraperitoneal administration. There have been no reports of significant adverse events associated with the use of **MM129**, confirming that this chemical has a positive safety profile in mice. At an anticancer-effective dose of 10 µM/kg, it was not lethal or harmful to mice, and it exhibited no toxicity [35].

Another new pyrazolo[4,3-*e*]tetrazolo[1,5-*b*][1,2,4]triazine sulfonamide (**MM131**) demonstrated promising anticancer potential. It exhibited an inhibitory effect on the viability and proliferation of the DLD-1 and HT-29 cells. The stimulation of both extrinsic and intrinsic apoptotic pathways is thought to be the molecular mechanism of action for this compound. This is associated with increased activity of caspase-8 and caspase-9 enzymes. Inhibition of key proteins involved in the progression and metastasis of colorectal cancer, such as sICAM-1, cathepsin B, and mTOR kinase, following incubation of cells with **MM131,** was observed. After 24 h of incubation, **MM131** was able to significantly lower mTOR concentrations in both colon cancer cell lines as compared to untreated cells, and the inhibitory impact was significantly larger than that of the reference drug. In addition, sICAM-1, which is elevated in the sera of patients with various malignancies and is thought to be a prognostic marker in patients with colorectal cancer, is an important molecule that is reduced following exposure to **MM131**. Moreover, it reduced cathepsin B implicated in enhanced invasion and angiogenesis of cancer cells, while at the same time increasing levels of beclin with tumor-suppressing properties [36].

**Figure 1:**
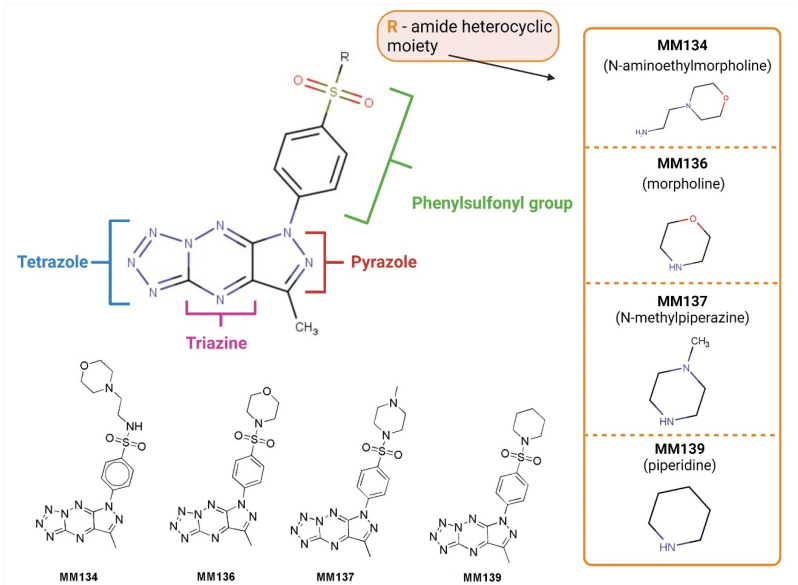
Chemical structure of investigated sulfonamides **MM134**, **MM136**, **MM137**, and **MM139**. Created with Biorender.com.

In this paper, we present preparation of new pyrazolo[4,3-*e*]tetrazolo[1,5-*b*][1,2,4]triazine sulfonamide derivatives (Figure 1) and their anticancer activity including cytotoxic and proapoptotic potential. To estimate the cytotoxic effect of tested compounds, the MTT assay was used [37].

Moreover, in our research, we focused on the detection of apoptosis by membrane alterations (externalization of phosphatidylserine on the outer plasma membrane of apoptotic cells). Double staining with acridine orange and ethidium bromide was additionally applied to visualize the morphological features of apoptosis and/or necrosis in cancer cells incubated with tested compounds. The changes in the mitochondria membrane potential (MMP; ΔΨm) following incubation with tested compounds were assessed with MitoTracker Red [38] as an indicator of mitochondrial function and apoptosis activation [39,40].

Additionally, using molecular docking and molecular dynamics techniques, selectivity for molecular targets with an established role in cancer pathogenesis was evaluated. Molecular docking results and predicted physicochemical parameters of the tested compounds were compared with the results obtained for the **MM129** and **MM131** derivatives that have been previously described in the literature [26,35,36].

## 2. Results

### 2.1. Chemistry

**Figure 2:**
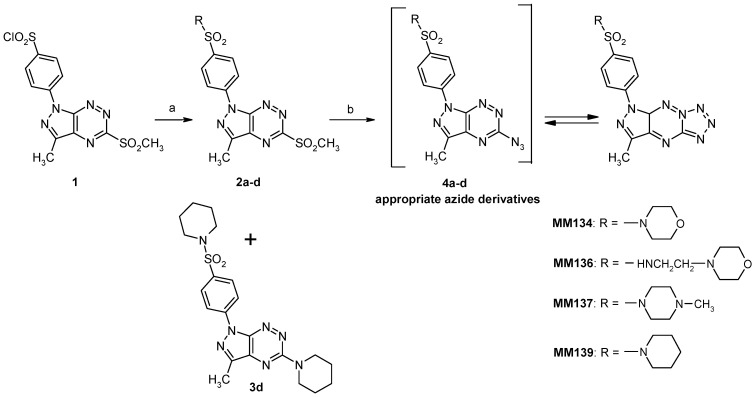
Reagents and conditions: (a) appropriate amine, MeCN, rt, overnight; (b) NaN_3_, EtOH, reflux.

The multi-step preparation of compound **1**, which is a crucial intermediate for the synthesis of the pyrazolo[4,3-*e*]tetrazolo[1,5-*b*][1,2,4]triazine sulfonamides (**MMs**), has been described in detail in our earlier work [41]. Chlorosulfone **1** readily reacts with cyclic amines to give the corresponding sulfone **2a-d** which undergo nucleophilic substitution reaction with sodium azide to provide the final tricyclic derivatives of the pyrazolo[4,3-*e*]tetrazolo[1,5-*b*][1,2,4]triazine ring system (**MM-sulfonamides**). Sulfonamide **3d** with a piperidine substituent in the C5 position was formed as a by-product of the synthesis of the corresponding sulfonamide **2d** (Figure 2). The NMR spectrum for **3d** lacks at δ = 3.63 ppm the characteristic singlet for the SO_2_CH_3_ group which is present on the NMR spectrum for the derivative **2d**. Instead of the singlet, there is a multiplet for the hydrogens of the piperidine ring in the range 1.6–1.8 ppm, which corresponds to 10 protons. The structure of all obtained compounds was confirmed by spectroscopic methods.

**Figure 3:**
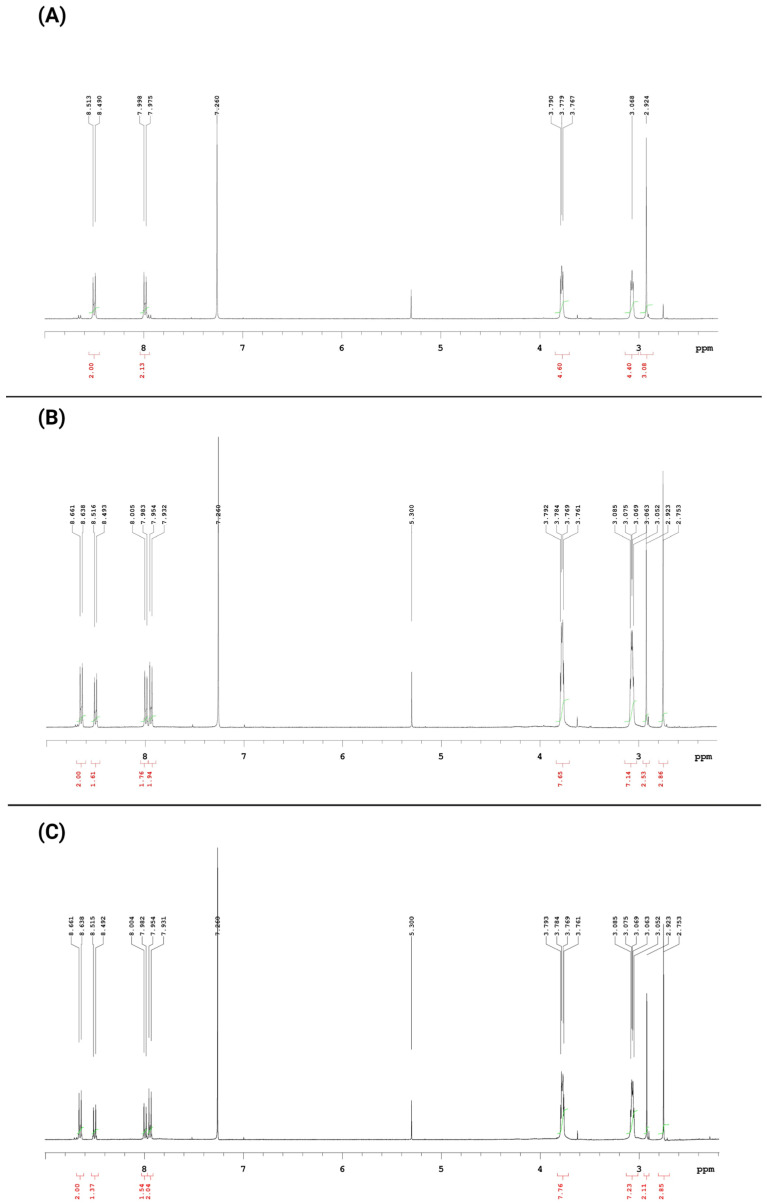
The ^1^H NMR spectrum (**A**) recorded immediately after solution of the compound **MM134** in deuterated chloroform and repeated (**B**) after 24 h and (**C**) after 48 h.

Many previous studies show that fused derivatives of the pyrazolo[4,3-*e*]tetrazolo[1,5-*b*][1,2,4]triazine in solution exist in tautomeric equilibrium with the corresponding 5-azido derivative of the pyrazolo[4,3-*e*][1,2,4]triazine ring system [25,36,41,42,43,44,45]. The ^1^H NMR spectrum recorded immediately after dissolution of the compound **MM134** in deuterated chloroform exhibited one singlet at δ = 2.92 ppm for CH_3_ group and two triplets at δ = 3.06 ppm and δ = 3.77 pp which can only relate to the morpholine ring and two doublets in aromatic region at 7.98 ppm and 8.50 ppm corresponding to protons in the phenyl ring of the tetrazole form **MM134** (Figure 3A). Moreover, there is one small singlet at 2.75 ppm and two very small doublets at 7.94 and 8.65 ppm that correspond to the methyl group and aromatic protons present in the appropriate azide form **4a**. The ^1^H NMR measurement for the same sample repeated after 24 h showed the shift of the tautomeric equilibrium toward the slight dominance of the azide form relative to tetrazole form (Figure 3B). Much more effective increase in the azide form was observed after 48 h and the ratio of the populations was 1:0.7 in favor of azide structure (Figure 3C). However, despite the partial decomposition of the investigated sample, the ^1^H NMR spectrum recorded after 2 weeks shows that in the solution again tetrazole derivative exists as the main form and the azide tautomer accounts for about 34%. A similar effect was observed for other presented tetrazole derivatives. It should be noted that these experiments showed that the tetrazole form was predominant after tautomeric equilibrium was established, which is consistent with earlier research results and literature data [42,43,44,45]. The ^1^H NMR spectra were also presented in Figure S1 of the SM1.PDF file in the Supplementary Material section.

### 2.2. Biological Studies

#### 2.2.1. MTT Assay

**Figure 4:**
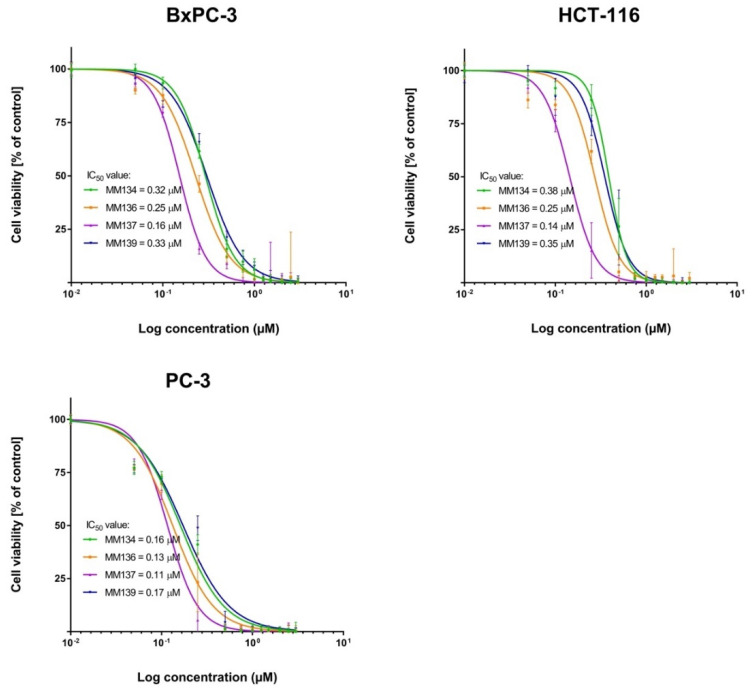
Determination of relative cancer cell viability of pancreas adenocarcinoma cell line (BxPC-3), colorectal carcinoma cell line (HCT-116) and prostate adenocarcinoma cell line (PC-3) treated with MM-compounds; ±SEM.

**Figure 5:**
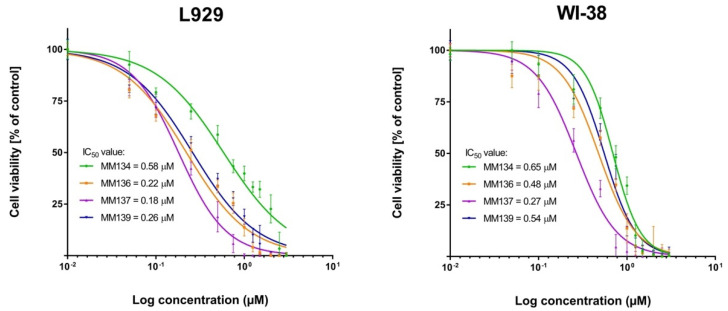
Determination of relative cell viability of mouse fibroblast cell line (L929) and human normal fibroblast cell line (WI-38) treated with MM-compounds; ±SEM.

The MTT assay used to determine cell viability showed that all tested novel pyrazolo[4,3-*e*][1,2,4]triazine-containing compounds possess cytotoxic activity towards cancer cell lines (BxPC-3, HCT-116, and PC-3) and, to a lesser extent, towards normal mouse (L929 cell line) and human (WI-38 cell line) fibroblasts. A reduction in cell viability after 72 h incubation with tested compounds was observed in all experimental series. The IC_50_ values obtained in the MTT assay are shown in Table 1. The cytotoxic effect was specific to cancer cells. Dose–response curves obtained from two independent experiments after 72 h exposure of cancer cells (BxPC-3, HCT-116, PC-3) and normal mouse (L929 cell line) and human (WI-39 cell line) fibroblasts to MM-compounds in shown in Figure 4 and Figure 5. The decrease in cell viability correlates with the increasing concentration of the compounds. The PC-3 cell line was more sensitive to the tested compounds than BxPC-3 and HCT-116 cells, which may indicate some degree of selectivity of the tested compounds for this cell line. MM-compounds exhibited more cytotoxic activity in the cancer cells than in normal mouse and human fibroblasts.

#### 2.2.2. Annexin V and Propidium Iodide Flow Cytometry Analysis

**Figure 6:**
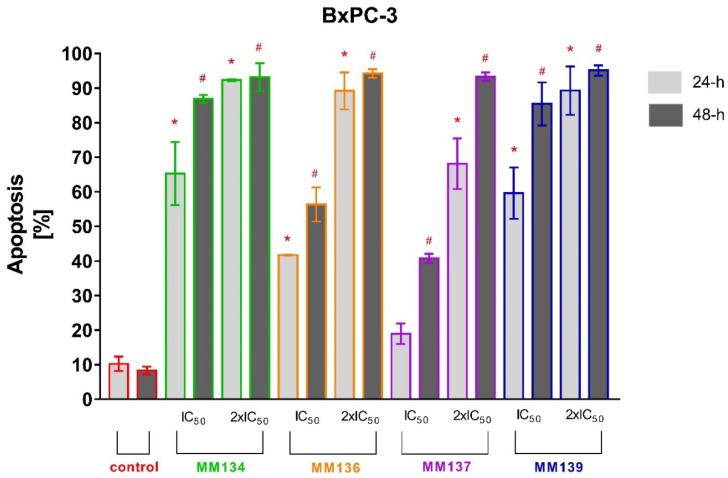
Determination of apoptosis induction in BxPC-3 cell line treated with IC_50_ and 2xIC_50_ concentrations of MM-compounds measured with Annexin V FITC. Data are presented as mean percentage of apoptotic cells (early and late apoptotic) ± SD values. The differences between the experimental samples and vehicle control were evaluated by the ANOVA test followed by Tukey’s test (*p* < 0.05). * significant difference compared to the negative control (24 h incubation time); # significant difference compared to the negative control (48 h incubation time); *p* < 0.05; *N* = 1 × 10^4^.

**Figure 7:**
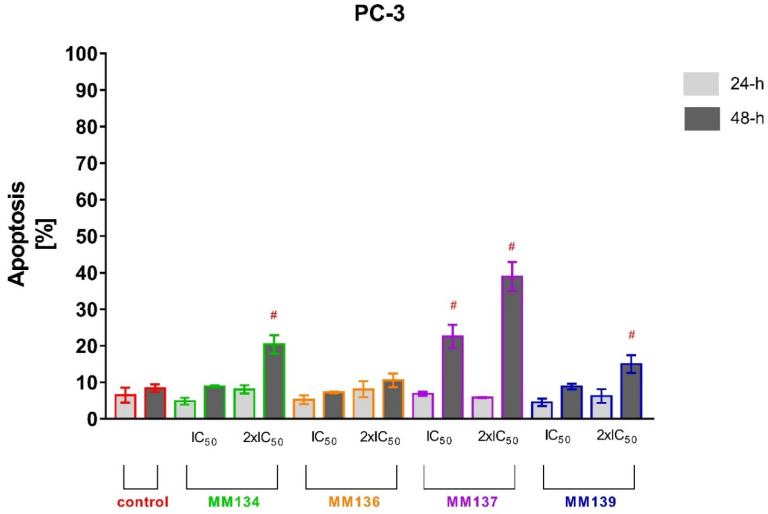
Determination of apoptosis induction in PC-3 cell line treated with IC_50_ and 2xIC_50_ concentrations of MM-compounds measured with Annexin V FITC following 24 and 48 h incubation time. Data are presented as mean percentage of apoptotic cells (early and late apoptotic) ± SD values. The differences between the experimental samples and vehicle control were evaluated by the ANOVA test followed by Tukey’s test (*p* < 0.05). # significant difference compared to the negative control (48 h incubation time); *p* < 0.05; *N* = 1 × 10^4^.

Tested cell lines that exhibited the most sensitivity to the cytotoxic effects of MM-compounds were selected for the study of their proapoptotic properties. The values obtained in the MTT test for the HCT-116 and BxPC-3 lines were very similar. It was decided that the pancreatic cancer line, which belongs to the neoplasms with a high mortality rate and a very aggressive course, will be used for further research. The apoptotic status of BxPC-3 and PC-3 cells after 24 and 48 h of incubation with triazine derivatives used in IC_50_ and 2xIC_50_ concentrations obtained in the MTT assay was determined utilizing flow cytometry using dual annexin V and propidium iodide staining (Figure 6 and Figure 7). Flow cytometry Supplementary Materials can be found in Figure S2 (BxPC-3 cell line) and Figure S3 (PC-3 cell line) of SM1.PDF file.

A very high increase in the apoptotic cell fraction was observed after 24 and 48 h incubation of BxPC-3 cells with 2xIC_50_ concentrations of all MM-compounds (from 68.1 ± 7.33% to 95.1 ± 1.48%), compared to the control group: 10.3 ± 2.09% (24 h incubation) and 8.3 ± 1.13% (48 h incubation).

The apoptotic response increased in order of **MM137** (18.9 ± 2.95%), **MM136** (41.7 ± 0.17%), **MM139** (59.6 ± 7.42%), and **MM134** (65.3 ± 9.1%) after 24 h incubation with IC_50_ concentrations of tested compounds and in analogous order of **MM137** (40.8 ± 1.36%), **MM136** (56.33 ± 4.96%), **MM139** (85.4 ± 6.25%), and **MM134** (86.9 ± 1.16%) after 48 h incubation. The number of necrotic cells following 24 and 48 h incubation of BxPC-3 cells with MM-compounds did not exceed 10% in all experimental series.

For 24 h incubation of PC-3 cells, no statistically significant increase in % of apoptotic cells was observed compared to negative control (6.5 ± 2.05%). In contrast, **MM134** (20.37 ± 2.5%), **MM137** (38.93 ± 4%), and **MM139** (15 ± 2.42%) used in 2xIC_50_ concentrations induced a statistically significant increase in mean percentage of apoptotic cells compared with negative control (8.37 ± 1.06%) after 48 h incubation of PC-3 cells. Moreover, a statistically significant increase in apoptosis was observed after 48 h incubation of cells with **MM137** (22.57 ± 3.18%) used in IC_50_ concentration. No statistically significant differences were observed in other experimental series after 48h incubation. The number of necrotic cells following 24 and 48 h incubation of PC-3 cells and BxPC-3 with MM-compounds did not exceed 10% in all experimental series. The obtained results indicate that the BxPC-3 cancer cells were more sensitive to the proapoptotic activity of the tested compounds used in both concentrations than the PC-3 cell line. Additionally, the **MM134** and **MM139** compounds had the most profound pro-apoptotic effects on BxPC-3 cancer cells.

#### 2.2.3. Dual Acridine Orange/Ethidium Bromide (AO/EB) Fluorescent Staining

**Figure 8:**
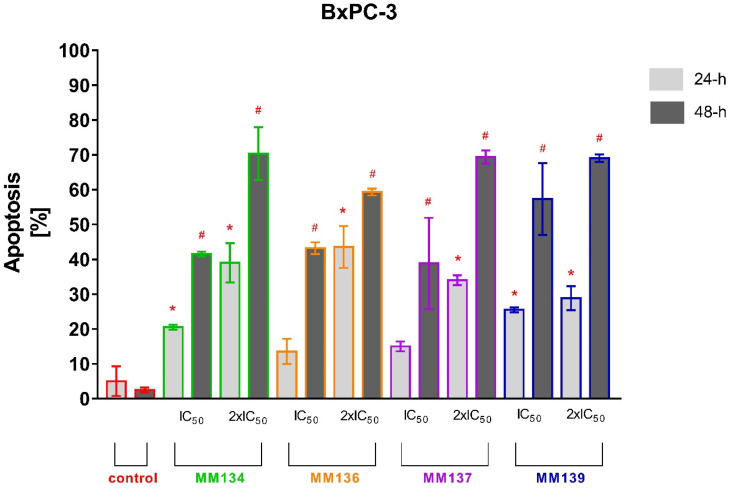
Determination of apoptosis induction in PC-3 cell line treated with IC_50_ and 2xIC_50_ concentrations of MM-compounds measured with AO/EB double staining following 24 and 48 h incubation time. Data are presented as mean percentage of apoptotic cells (early and late apoptotic) ± SD values. The differences between the experimental samples and vehicle control were evaluated by the ANOVA test followed by Tukey’s test (*p* < 0.05). * significant difference compared to the negative control (24 h incubation time); # significant difference compared to the negative control (48 h incubation time); *p* < 0.05; *N* = 200.

**Figure 9:**
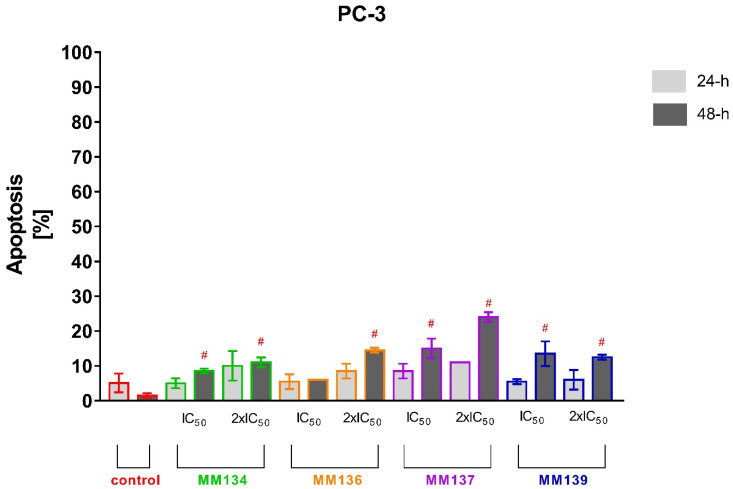
Determination of apoptosis induction in PC-3 cell line treated with IC_50_ and 2xIC_50_ concentrations of MM-compounds measured with AO/EB double staining following 24 and 48 h incubation time. Data are presented as mean percentage of apoptotic cells (early and late apoptotic) ± SD values. The differences between the experimental samples and vehicle control were evaluated by the ANOVA test followed by Tukey’s test (*p* < 0.05). # significant difference compared to the negative control (48 h incubation time); *p* < 0.05; *N* = 200.

**Figure 10:**
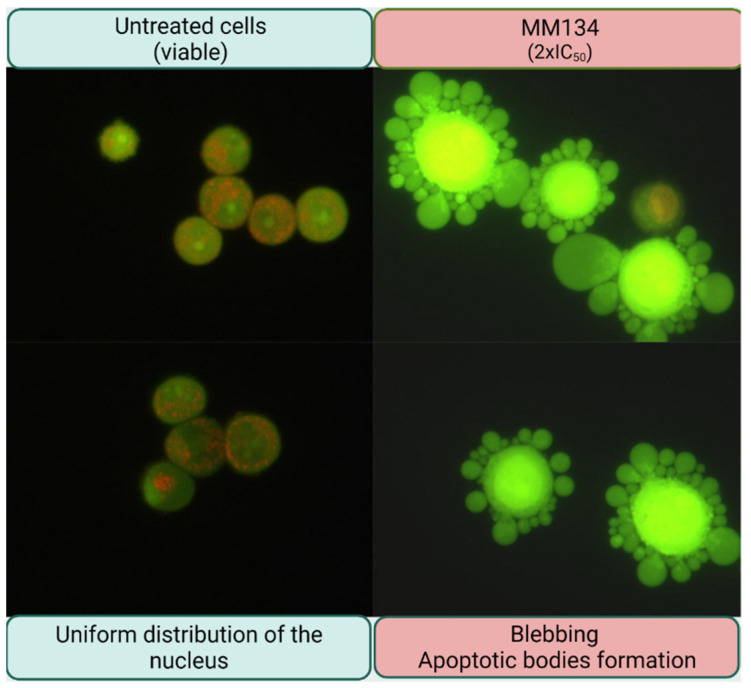
Typical morphological features of apoptosis in BxPC-3 following treatment with **MM136** compound. Characteristic membrane blebbing can be observed in cancer cells after the treatment with the tested compound in 2xIC_50_ concentration compared with cells from control group in which cells are rounded and exhibit uniformly distributed nucleus. Cells were visualized under a fluorescence microscope OLYMPUS BX60, magnification 40×.

Apoptosis induction estimated by flow cytometry analysis was confirmed with acridine orange/ethidium bromide staining (Figure 8 and Figure 9). This method combines the differential uptake of fluorescent DNA-binding dyes AO and EB with the morphologic aspect of chromatin condensation in the stained nucleus, allowing one to distinguish viable, apoptotic, and necrotic cells (Figure 10) [46].

Similarly to the flow cytometry results, a high increase in the apoptotic cell fraction was observed after 24 and 48 h incubation of BxPC-3 cells with 2xIC_50_ concentrations of all MM-compounds (from 28.8 ± 3.46% to 70.35 ± 7.57%), compared to the control group: 5 ± 4.24% (24 h incubation) and 2.5 ± 0.7% (48 h incubation). **MM134** and **MM139** used in both tested concentrations induced a statistically significant increase in the apoptotic cell fraction following 24 and 48 h incubation of BxPC-3 cells. Only **MM136** and **MM137** used in IC_50_ concentrations did not induce a significant increase in apoptotic fraction following 24 h incubation of BxPC-3 cells with the compounds.

No statistically significant increase in apoptosis % was detected following 24 h incubation of PC-3 cells with tested compounds. **MM134**, **MM137**, and **MM139** induced a statistically significant increase in % of apoptotic cells in the PC-3 line following 48 h incubation compared to control group (1.5 ± 0.7%) in both tested concentrations. The mean % of apoptotic cells did not exceed 30% following 48 h incubation with tested compounds and were: **MM134** IC_50_ (8.5 ± 0.7%), **MM134** 2xIC_50_ (11 ± 1.41%), **MM136** 2xIC_50_ (14.5 ± 0.7%), **MM137** IC_50_ (15 ± 2.82%), **MM137** 2xIC_50_ (24 ± 1.41%), **MM139** IC_50_ (13.5 ± 3.53%), and **MM139** 2xIC_50_ (12.5 ± 0.7%). Again, a stronger apoptotic response was observed in BxPC-3 cells following 24 and 48 h incubation with the tested compounds.

#### 2.2.4. Changes in Transmembrane Mitochondrial Potential-MitoTracker Red

**Figure 11:**
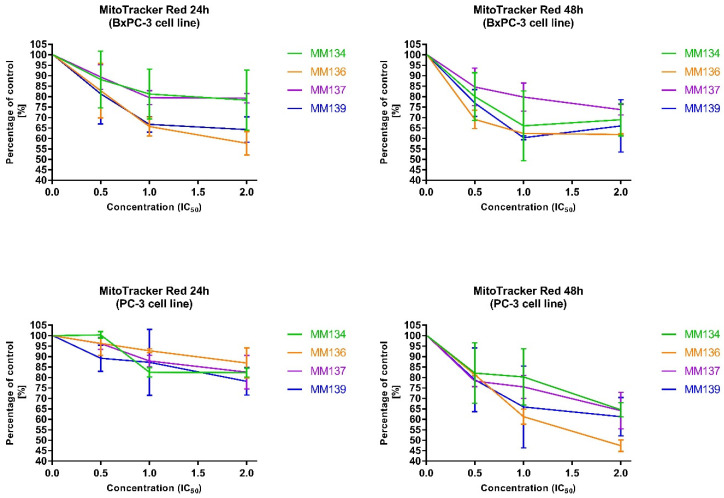
Changes in the mitochondria membrane potential (MMP) after 24 h and 48 h incubation with MM-compounds in all tested cancer cell lines; ±SD value.

The fitness of the mitochondria and changes in MMP were assessed using MitoTracker Red. Data for experimental groups were presented as mean percentage of control group fluorescence intensity ± SD value (Figure 11, Table 2).

After 24 and 48 h incubation of BxPC-3 and PC-3 cells with tested compounds used in concentrations followed by IC_50_ values (0.5xIC_50,_ IC_50_, and 2xIC_50_), a decrease in fluorescence intensity reflecting a reduction in MMP was observed. Following 24 and 48 h incubation of BxPC-3 cells with tested compounds used in 2IC_50_ concentrations, MMP decreased with an increase in compound concentration in the order of **MM137, MM134, MM139** and **MM136**. For the 48 h incubation of BxPC-3 cell lines, a decrease in MMP was observed after use of **MM134** and **MM139** compounds in 0.5xIC_50_ and IC_50_ concentrations with an increase in MMP following incubation with 2xIC_50_ concentrations. After both 24 and 48 h incubation times, the **MM136** used in 2xIC_50_ concentrations induced the highest reduction in MMP (% of control were: 57.67 ± 5.56% and 61.83 ± 0,46% for 24 h and 48 h incubation, respectively).

The 24 and 48 h incubation of PC-3 cells also led to a decrease in MMP. **MM139** (78.14 ± 6.56%) and **MM136** (47.38 ± 2.74%) used in the concentration of 2xIC_50_ induced the highest reduction in MMP among the MM-compounds in PC-3 cells after 24 and 48 h incubation, respectively.

### 2.3. Data Analysis

#### 2.3.1. MTT Assay

A statistical program (*Graphpad Prism 7*) was used to analyze obtained data (MTT test). The dose–response analysis was performed to estimate the inhibitory concentration (IC_50_) of the tested compounds. The IC_50_ value is defined as a concentration of the tested compound that leads to a reduction in cell pool viability by 50% compared to the negative control (accepted as 100%).

$$\%\;cell\;viability=\frac{\left(Absorbance\;value\;of\;treated\;cells-Absorbance\;value\;of\;blank\right)}{\left(Absorbance\;value\;of\;untreated\;cells-Absorbance\;value\;of\;blank\right)}\times 100\%$$

#### 2.3.2. Apoptosis Detection

Data are presented as mean percentage of apoptotic cells (early and late apoptotic) ± SD values. The differences between the experimental samples and vehicle control were evaluated by the ANOVA test followed by Tukey’s test (*p* < 0.05). Experiments were performed in triplicate. For flow cytometry analysis with Annexin V-FITC staining, the approximate number of analyzed cells was (*N* = 1 × 10^4^), contrasting with the dual acridine orange/ethidium bromide (AO/EB) fluorescent staining (*N* = 200).

### 2.4. Computational Studies

#### 2.4.1. Molecular Docking Simulation

The PDB codes for the downloaded proteins were 4wa9 [47], 2xyn [48], 3mvh [49], 3d0e [50], 2x18 [51], 5n7e [52], 3gen [53], 6qn5 [54], 6qnl [54], 3bhu [55], 2w9z [56], 5l2s [57], 1ua2 [58], 5mza [59], 6bcx [60], 6zwo [61], 7bea [62] for ABL1, ABL2, AKT1, AKT2, AKT3, BCR, BTK, CA-IX, CA-XII, CDK2, CDK4, CDK6, CDK7, ICAM-1, mTOR1, mTOR2 and PD-L1, respectively. Downloaded macromolecular targets were prepared for molecular docking simulation by addition of hydrogens, removal of redundant water molecules, computing Gasteiger charge, and assigning Autodock-4 (AD4) [63,64] atom type followed by saving them in the default autodock format PDBQT [65,66].

All the rotatable, non-rotatable, and un-rotatable bonds were assigned in all the six triazine sulfonamide derivatives, followed by saving their structures in the protein databank (PDB) format.

A suitable grid box covering all the extending conformations of the complexed reference ligands as well as the majority of the interacting macromolecular residues were formed for the examined molecular targets. The grid parameters for each of the targets were saved in a respective grid parameter file (GPF) for each anticancer target to be utilized by the Autogrid utility of the Autodock suite for the generation of map files required for performing molecular docking simulations. The grid parameters for each of the anticancer targets used in this study are tabulated in Table 3.

The map files for various atom types of the macromolecular target as well as ligands generated by the Autogrid utility were utilized by the Autodock software to perform the molecular docking simulation. The molecular docking simulation process for each macromolecular target was validated by considering the chemical resemblance as well as the overlay of the docked conformation of the ligand regarding its bioactive conformation [67,68].

Once the molecular docking simulation process was validated by considering the above-stated parameters, similar parameters were utilized to perform the simulation studies of the newly designed triazine sulfonamide analogs.

Molecular docking simulation-based screening of the designed triazine analogs against the anticancer drug targets, which are actively involved in the pathophysiology of human cancers, revealed that the molecules **MM136** and **MM139** were the most potent inhibitors of all the anticancer targets considered in the current experimental study [66,69,70,71]. The binding scores of four new triazine sulfonamide analogs and previously studied compounds (**MM129** and **MM131**) as well as all the reference ligands for all the macromolecular targets are shown in Table 4.

**MM134** was found to inhibit AKT2 (binding energy of −11.81) the strongest of the tested compounds. Based upon the binding score obtained after performing molecular docking simulation-based virtual screening of **MM136** against all macromolecular anticancer targets, it was observed that the **MM136** molecule strongly interacts with AKT1, mTOR1, mTOR2, and PD-L1 (respective binding scores: −12.42, −9.08, −8.79, and −12.33), and is supposed to give anticancer activity through the inhibition of the given enzymes. **MM137** is the most potent inhibitor of ABL2 (binding score: −10.46) of all tested compounds. In contrast, **MM139** was found to be a potent inhibitor of nine of all seventeen macromolecular anticancer drug targets, i.e., ABL1, AKT3, BTK, CA-IX, CA-XII, CDK2, CDK4, CDK7, or ICAM-1, considered in the current study [72,73]. **MM134**, **MM136**, **MM137**, and **MM139** derivatives exhibited better molecular docking results than the previously described **MM129** and **MM131** derivatives in terms of binding to ABL2, AKT1, AKT2, BTK, ICAM-1, mTOR1, and PD-L1, while **MM129** exhibited the highest binding activity towards CDK6 (binding energy of −10.83) and CDK7 (binding energy of −10.04).

**Figure 12:**
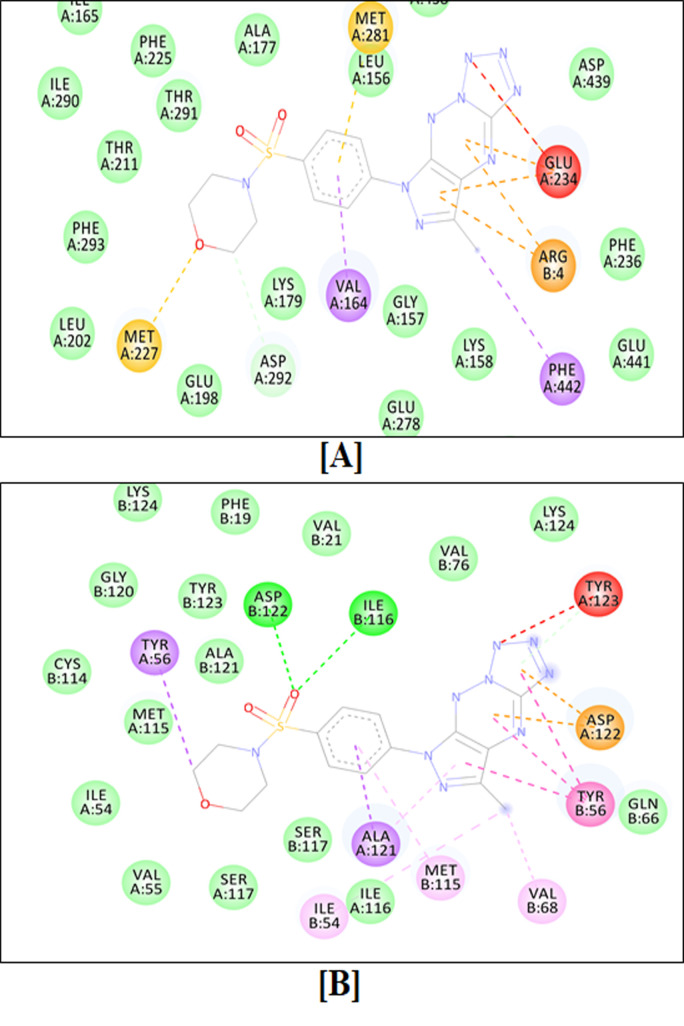
Two-dimensional binding interactions of the triazine sulfonamide analog **MM136** against human AKT1 (**A**) and PD-L1 (**B**).

**Figure 13:**
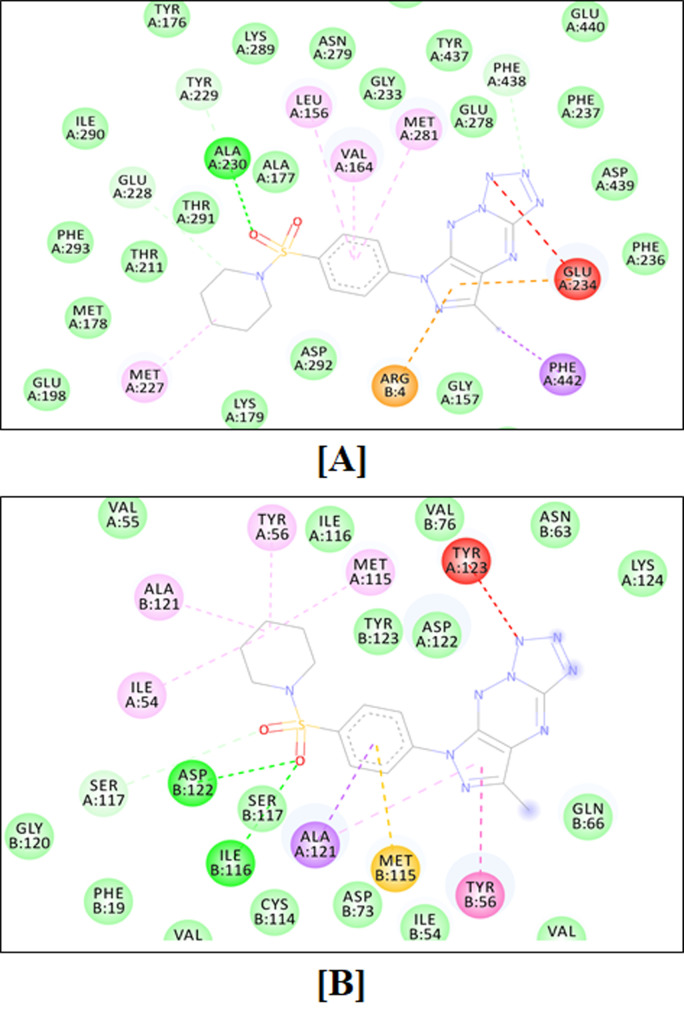
Two-dimensional binding conformation of the triazine sulfonamide analog **MM139** against human AKT1 (**A**) and PD-L1 (**B**).

The two-dimensional binding interactions of **MM136** against human AKT1 and PD-L1 are shown in Figure 12A,B, respectively, while the two-dimensional binding interactions of **MM139** against human AKT1 and PD-L1 are shown in Figure 13A,B, respectively.

#### 2.4.2. Molecular Dynamics Simulations

Molecular dynamics simulations revealed that the macromolecular complexes of ligands **MM136** and **MM139** against AKT1 as well as PD-L1 enzymes were found to be most stable throughout the simulation time concluding that both of these ligands are supposed to be potent anticancer agents and their therapeutic effect is executed via synergistic targeting of both the AKT1 and PD-L1 enzyme.

The target enzyme AKT1 has 300 residues distributed in a macromolecular chain consisting of 2450 heavy atoms out of a total of 4885 atoms. The macromolecular target has 40% of secondary structures in the form of 26% of alpha helices and 17% of beta-strands, which were found to be conserved during the simulation process. **MM136** possesses 28 heavy atoms out of a total of 43 atoms. Dynamic simulation of the macromolecular complex of **MM136** against the AKT1 target clearly showed that the root mean square deviation (RMSD) for the fluctuation of the protein backbone was in-between 1.2 and 1.8 Å which is well within the acceptable range. Similarly, the ligand **MM136** showed some initial fluctuations up to 20 ns while making certain moves within the active site to achieve the stable conformation followed by its stabilized vibrations within the range of 1.8–2.4 Å. Afterward, the complexed ligand had attained the most stable conformation and remained stable throughout the simulation process with very little fluctuation. The root mean square fluctuation (RMSF) of the macromolecular backbone was found to be well within the range of 0.9–1.2 Å throughout the simulation process. Macromolecular residues such as Val164, Ala177, Ala230, Met281, Asp292, Asp439, and Phe442 of AKT1 were found to be interacting with the ligand **MM136** throughout the simulation process.

**MM139** has 28 heavy atoms out of a total of 47 atoms. Dynamic simulation of the macromolecular complex of **MM139** against AKT1 clearly showed RMSD in-between 1.5 and 2.8 Å which is well within the acceptable range of 3 Å. Similarly, the ligand **MM139** showed minimal fluctuations throughout the simulation process and remained stable within the 0.8–1.8 Å range throughout the simulation process. The RMSF value observed for the AKT1 macromolecule was found to be in-between 0.8 and 2.0 while the RMSF for **MM139** was found to be within 1.0–2.1 Å throughout the simulation process. Residues such as Leu156, Phe161, Ala177, Ala230, Glu234, Met281, Tyr437, Phe438, Asp439, and Phe442 of AKT1 were found to be interacting with the ligand **MM139**.

The target enzyme PD-L1 has 249 residues distributed in two macromolecular chains consisting of 1906 heavy atoms out of a total of 3791 atoms. The macromolecular target has 40% of secondary structures in the form of 2% alpha helices and 39% of beta-strands, which were found to be conserved during the simulation process. **MM136** possesses 28 heavy atoms out of a total of 43 atoms. Dynamic simulation of the macromolecular complex of **MM136** has clearly shown that the RMSD for the fluctuation of the protein backbone was in-between 1.8 and 4.8 Å which is well within the acceptable range of 3 Å. Similarly, the ligand **MM136** showed some little fluctuations in-between the range of 1.8 Å in the initial 10 ns timeframe, while making certain moves within the active site to achieve the stable conformation. Afterward, the complexed ligand had attained the most stable conformation and remained it throughout the simulation process with very little fluctuation. Except for a few residues, the RMSF of the macromolecular backbone was found to be well within the range of 0.8–2.4 Å. The two macromolecular residues showing the RMSF value up to 5.6 Å were found, which is common in all the simulation processes. The RMSF value observed for **MM136** was found to be within 0.5–1.5 Å throughout the simulation process. Macromolecular residues such as Ile54, Tyr56, Met115, Ser117, Ala121, Asp122, and Tyr123 of chain-A as well as Tyr56, Val68, and Tyr123 of chain-B of PD-L1 were found to be interacting with the ligand **MM136** throughout the simulation process.

**MM139** has 28 heavy atoms out of a total of 47 atoms. Dynamic simulation of the macromolecular complex of **MM139** has clearly shown RMSD in-between 2.4 and 4.8 Å which is well within the acceptable range of 3 Å. Similarly, the ligand **MM139** showed minimal fluctuations throughout the simulation process and remained stable within the 1.0–3.2 Å range. The RMSF value observed for **MM139** was found to be within 1.5–2.2 Å throughout the simulation process. Residues such as Ile54, Tyr56, Ala121, Asp122, Tyr123, and Lys124 of chain-A as well as Ile54, Tyr56, Ser117, and Tyr123 of chain-B of PD-L1 were found to be interacting with the ligand **MM139**.

Dynamics simulations of the macromolecular complexes of **MM136** as well as **MM137** against both AKT1 and PD-L1 target enzymes revealed that both of the ligands were interacting strongly within the active site of the target enzyme and that both the target enzymes as well as complex ligands remained stable throughout the simulation process. The dynamic simulation of MM136 and MM139 complexed with AKT1 enzyme has shown strong binding interactions of the complexed ligand with adequate stability observed during the simulation process when compared with the macromolecular complex of ligands **MM136** and **MM139** against PD-L1 enzyme.

The computational results have revealed that these compounds are found to be potent anticancer agents and they are supposed to execute their therapeutic effect by interacting with the human AKT1 and PD-L1 enzymes.

The detailed reports of molecular dynamics simulation of MM-compounds can be found in Supplementary Materials for **MM136** with AKT1 (SM2.PDF) and PD-L1 (SM3.PDF), **MM139** with AKT1 (SM4.PDF) and PD-L1 (SM5.PDF) and **MM137** with PD-L1 (SM6.PDF).

#### 2.4.3. Drug Likeness and ADMET

Swiss-ADME (http://www.swissadme.ch, accessed on 1 May 2022) [74] was used to estimate drug likeness and ADMET properties of MM-compounds. The bioavailability of compounds is estimated based on five critical parameters indicating drug lipophilicity, size, polarity, insolubility, flexibility, and insaturation. The optimal range for each property is as follows: flexibility (no more than nine rotatable bonds), lipophilicity (XLOGP3 between −0.7 and +5.0), size: molecular weight (MW between 150 and 500 g/mol), polarity (topological polar surface area (TPSA) between 20 and 130 Å2), solubility (LogS not higher than 6), and saturation (fraction of carbons in the sp3 hybridization (Csp3) not less than 0.25). The tested compounds may not be orally bioavailable due to high polarity and TPSA, considering sulfur and phosphorus as polar atoms (Table 5). Tested MM-compounds may not be orally bioavailable due to high polarity (too high TPSA values; TPSA > 130 Å2).

The tested compounds showed no BBB permeability. Moreover, derivatives may constitute the substrate for glycoprotein P (PGP) that belongs to ABC-transporters that restrict the compounds from entering the central nervous system and are involved in tumor multidrug resistance. MM137 and MM139 showed good overall HIA.

50 to 90% of therapeutic molecules are the substrates of isoforms of cytochrome P450, including CYP2D6 and CYP3A4. The MM137 molecule possesses the best pharmacokinetic parameters, including high GI absorption and no inhibitory activity against CYP enzymes. The ADMET properties predicted by using Swiss-ADME (http://www.swissadme.ch, accessed on 1 May 2022) [74] are tabulated in Table 6.

Again, MM137 showed the best drug-likeness properties with one violation according to the Lipiński rule (number of nitrogen or oxygen atoms (NorO) > 10) (Table 7).

## 3. Discussion

The MTT assay used to determine cell viability showed that all tested MM-compounds possess cytotoxic activity towards cancer cell lines (BxPC-3, HCT-116, PC-3) and to a lesser extent towards the normal mouse fibroblast (L929) cell line and human fibroblasts (WI-38 cell line). The cytotoxic effect was specific for cancer cells. The MM137 compound exhibited the highest cytotoxic activity with IC_50_ values in cancer cells ranging from 0.11 to 0.16 μM. The highest difference between the cytotoxic activity of compounds between cancer and normal cells was observed for the MM134 compound, where the compound exhibited 4 times higher cytotoxic activity in the PC-3 cell line compared to normal human fibroblasts (WI-38 cell line).

In our study, the activation of apoptosis was detected after 24 and 48 h exposure of BxPC-3 and PC-3 cells to tested compounds in IC_50_ and 2xIC_50_ concentrations. Tested compounds were used in concentrations followed by IC_50_ values to establish whether the cytotoxic effects were due to apoptosis induction. **MM134**, **MM136**, **MM137**, and **MM139** induced increase in number of apoptotic cells both in BxPC3 and PC-3 cell lines; however, the effect was more profound in the BxPC-3 cell line where tested compounds induced apoptosis of 18.9–65.3% cells for 24 h incubation with tested compounds in IC_50_ values, and 40.8–86.9% following 48 h incubation with the derivatives as indicated by flow cytometry analysis using annexin V-FITC staining. The percentage of apoptotic cells following incubation of BxPC-3 cells with tested compounds increased in order of **MM137, MM136, MM139** and **MM134.**

No statistically significant increase in the number of apoptotic cells was observed following 24 h incubation of PC-3 cells with IC_50_ or 2xIC_50_ concentrations of MM-compounds. In contrast, **MM134** (20.37 ± 2.5%), **MM137** (38.93 ± 4%), and **MM139** (15 ± 2.42%) used in 2xIC_50_ concentrations induced as statistically significant increase in mean percentage of apoptotic cells compared with negative control after 48 h incubation of PC-3 cells.

Similar results were obtained with acridine orange/ethidium bromide double staining; however, the mean percentage of apoptotic cells was lower than this indicated by annexin V-FITC staining. This may be due to the large sample size in annexin V-FITC analysis (10,000 cells) compared with 200 cells analyzed in the acridine orange/ethidium bromide double staining. Moreover, annexin V-FITC cytometry analysis uses automate cell sorting, and therefore remains a more reliable method of apoptosis detection than manual counting and identification of apoptotic cells using fluorescence microscopy. Despite that, the apoptosis induction was seen using acridine orange/ethidium bromide double staining.

It is one of the initial alterations linked with programmed cell death to observe a decrease in MMP. It is known that the mitochondrial membrane permeability increases during apoptosis. As a result, mitochondrial proteins such as cytochrome c and apoptosis-inducing factor (AIF) are released into the cytosol, leading to the activation of the intrinsic apoptosis pathway. After both 24 and 48 h incubation times, **MM136** used in the 2xIC_50_ concentration induced the highest reduction in MMP in the BxPC-3 cell line. **MM139** and **MM136** used in the concentration of 2xIC_50_ induced the highest reduction in MMP among MM compounds in PC-3 cells after 24 and 48 h incubation, respectively. However, the induction of intrinsic and extrinsic apoptosis pathways needs to be elucidated.

Based on previously identified potential targets of pyrazolo-triazine derivatives (**MM129**, **MM131**), we performed molecular docking to identify the most potent compound among the newly designed pyrazolo[4,3-*e*]tetrazolo[1,5-*b*][1,2,4]triazine sulfonamides (**MM134, MM136, MM137** and **MM139**) and to establish the most probable molecular mechanism of these molecules. For this purpose, we estimated the binding capacity of the tested MM-compounds to a multitude of molecular targets with established roles in cancer: ABL1, ABL2, AKT1, AKT2, AKT3, BCR, BTK, CA-IX, CA-XII, CDK2, CDK4, CDK6, CDK7, ICAM-1, mTOR, mTOR2, and PD-L1. **MM134** was found to be the most potent inhibitor of AKT2, while **MM136** strongly bound to AKT1, mTOR1, mTOR2, and PD-L1. In contrast, **MM139** was found to be the most potent inhibitor (of all MM-compounds included in the docking screening) of nine of seventeen targets evaluated in this study, including: ABL1, AKT3, BTK, CA-IX, CA-XII, CDK2, CDK4, CDK7 and ICAM-1. **MM134, MM136, MM137,** and **MM139** exhibited better binding scores in dockings towards ABL2, AKT2, BTK, ICAM-1, mTOR1 and PD-L1, than **MM129** and **MM131** compounds. **MM129** exhibited the highest binding activity towards CDK6 and CDK7 enzymes. This may suggest that **MM134, MM136, MM137** and **MM139** compounds may exert biological activity through the inhibition of the AKT-mTOR pathway, while **MM129** and **MM131** may exhibit antiproliferative potential through their binding with CDK enzymes. Moreover, **MM134**, **MM136**, **MM137**, and **MM139** compounds were shown to strongly bind to AKT1 and PD-L1. The stability of complexes formed between macromolecule targets and ligands preselected in a docking procedure was assessed during molecular dynamics simulation. The macromolecular complex of AKT1 with **MM136** and **MM139** was found to be highly stable throughout the simulation timeframe of 100 ns. This clearly indicates the inhibitory potential of **MM136** and **MM139** against the AKT1. The molecular dynamics simulation also revealed that **MM136** and **MM139** compounds bind to the PD-L1 active site and form the most stable complexes. The comparative analysis of the molecular dynamic simulation of AKT1 and PD-L1 against **MM136** and **MM139** indicates that these ligands are found to be more stable while interacting with the AKT1 target as compared with PD-L1. These results indicate that **MM134, -6, -7,** and **-9** may work as potential dual AKT-mTOR and PD-L1 inhibitors, with **MM136** exhibiting the best in silico results.

Furthermore, in in vivo studies, **MM129** exhibited a good pharmacokinetics profile with rapid absorption and bioavailability of 68.6% following intraperitoneal administration. Therefore, we decided to compare the computationally estimated pharmacokinetic properties of **MM129** and **MM131** with **MM134, MM136, MM137,** and **MM139** compound profiles using the Swiss-ADME web tool (http://www.swissadme.ch, accessed on 1 May 2022) [74]. We have shown that tested compounds may exhibit better bioavailability than **MM129** and **MM131**. Moreover, **MM137** and **MM139** showed good gastrointestinal absorption in silico. Furthermore, **MM137** showed no inhibitory activity against CYP enzymes and the best drug likeliness properties of all compounds. This may limit potential drug–drug interactions in the living organisms [75,76,77]. However, all tested derivatives included in the study were shown not to be BBB-permeable and may work as substrates for the PGP enzyme, limiting their use in the central nervous system, and this may restrict their antitumor effects [78,79]. These estimates need confirmation in the following in vivo studies.

**Figure 14:**
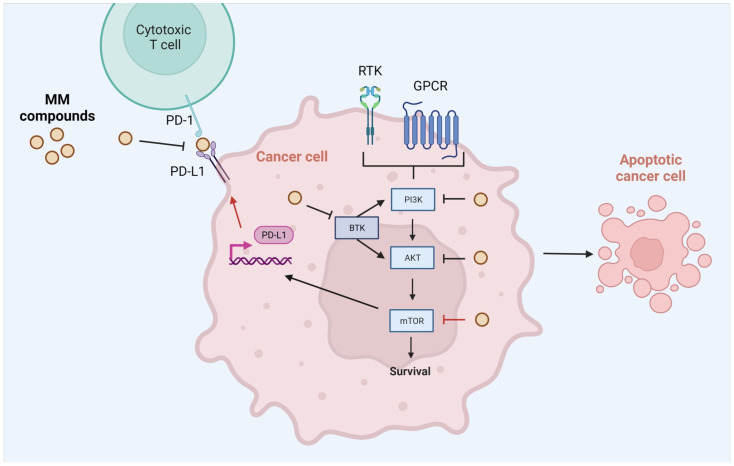
Probable mechanism of action of pyrazolo[4,3-*e*]tetrazolo[4,5-*b*][1,2,4]triazine sulphonamides (MM-compounds). Abbreviations: AKT—protein kinase B BTK—Bruton’s tyrosine kinase; JAK—tyrosine-protein kinase JAK; mTOR—mammalian target of rapamycin; PI3K—phosphoinositide 3-kinases; PD-1—Programmed cell death protein 1; PD-L1—Programmed death-ligand 1. Created with Biorender.com.

Many questions regarding the MM derivatives remain unanswered. First, the reduction in MMP levels may indicate the activation of an intrinsic pathway of apoptosis. However, further investigations, e.g., exploration of caspase levels associated with activation of intrinsic and extrinsic apoptosis pathways, need to be performed. Studies of **MM137** derivative in DLD-1 and HT-29 cell lines suggest the activation of both apoptosis pathways [41]. The inhibitory activity of compounds associated with the AKT-mTOR pathway and the PD1-PD-L1 association needs to be confirmed in screening assays and biological systems in in vivo conditions. The exploration of biological activity of other analogs of MM derivatives may lead to optimization and discovery of novel, safe and potent inhibitors of AKT-mTOR or PD1-PD-L1 interaction. The probable mechanism of action of **MM134**, **MM136**, **MM137**, and **MM139** compounds is shown in Figure 14.

Moreover, it has been found that the addition of 5-FU to **MM129** exhibited a synergistic effect on cancer cells with reduced PD-L1 mRNA and protein levels in DLD-1 and HT-29 cell lines [26]. Previously, Kim et al., reported that AKT activation may lead to the development of resistance to 5-FU [80]. In accordance with these findings, the use of **MM129** with 5-FU led to a decrease in phosphorylation of AKT compared with 5-FU alone [26]. According to a study conducted by Lastwika et al., the PI3K/AKT/mTOR pathway is involved in the formation of tumor cell immune resistance. mTOR kinase activation is substantially related to the surface expression of the tumor suppressor protein PD-L1 in human lung cancer cells, and the stimulation of the AKT/mTOR axis facilitates immune evasion through increased PD-L1 expression. PI3K/Akt/mTOR inhibition restricts cell proliferation, migration, and survival, at the same time facilitating immune surveillance and immune-cell-dependent tumor cell killing [81].

Increased expression of PD-L1 is a critical mechanism by which tumor cells are able to evade T-cell immunity. According to emerging evidence, chemotherapeutic drugs can affect the expression of PD-L1 on cancer cells, which may have an influence on immune evasion. Human colon cancer cell lines usually do not express the PD-L1 protein on their cell surfaces, but treatment with 5-FU induces its expression significantly. Therefore, the combination of drugs may enhance the effectiveness of colon cancer treatment [82]. AKT/PI3K activation can boost PD-L1 expression by increasing extrinsic signaling or decreasing the expression of negative regulators such as phosphatase and tensin homolog (PTEN). It is possible that downregulation of PTEN may result in the activation of PI3K/AKT, which will then facilitate the production of PD-L1. Furthermore, blocking the PD-1/PD-L1 pathway in gastrointestinal stromal tumors can reduce the apoptosis of CD8+ T cells by governing the PI3K/AKT/mTOR pathway. In addition, it has been found that the overexpression of PD-L1 in colorectal cancer cells may result in the activation of the PI3K/AKT pathway [83]. Moreover, inhibition of mTOR kinase may enhance the expression of PD-L1 on cancer cell surfaces, contributing to the reduced efficacy of mTOR inhibitors. Therefore, the increased expression of PD-L1 in cancer cells may increase the availability of epitopes to which anti-PD-L1 antibodies may bind. Similarly, CDK4/6 inhibition may increase PD-L1 levels in cancer cells, making them substantially more sensitive to PD-1 blocking therapy [84]. Taken together, these findings of dual inhibition of AKT-mTOR or CDK4/6 and PD-L1 may constitute an important way of targeting cancer [85].

## 4. Materials and Methods

### 4.1. Chemical Synthesis

Melting points were determined on a Mel-Temp apparatus and were uncorrected. ^1^H and ^13^C NMR spectra were recorded on a Varian spectrometer (400 MHz for ^1^H and 100 MHz for ^13^C). The chemical shift values were expressed in ppm (part per million) with tetramethylsilane (TMS) as an internal reference. Molecular weight of final compounds was assessed by electrospray ionization mass spectrometry (ESI/MS) on an Agilent Technologies 6538 UHD Accurate Mass Q-TOF LC/MS (Agilent Technologies, Inc., Santa Clara, CA, USA). Elemental compositions were within ±0.4% of the calculated values. For preparation and spectroscopic data of compound **1,** see the literature [86].

#### 4.1.1. Synthesis of Sulfonamides (**2a**–**d**)

Derivative **1** (194 mg, 0.5 mmol) was dissolved in anhydrous acetonitrile (5 mL) and appropriate amine (1.75 mmol) was added. The reaction was stirred overnight at room temperature; then, the reaction mixture was concentrated in vacuo to afford the crude sulfonamide, as a yellow solid. The residue was purified on silica gel using a mixture of CH_2_Cl_2_:EtOH (25:1) as eluent to give the titled compounds as a yellow solid.

*N*-(2-morpholinoeth-1-yl)-4-(3-methyl-5-methylsulfonyl-1*H*-pyrazolo[4,3-*e*][1,2,4]triazyn-1-yl)benzenesulfonamide (**2a**).

Yield 77%. Melting point: 209–212 °C; ^1^H NMR (DMSO) δ: 2.80 (s, 3H), 2.93–2.96 (m, 4H), 3.63 (s, 3H), 3.64–3.67 (m, 4H), 8.06 (d, 2H, J = 8.8 Hz), 8.63 (d, 2H, J = 8.8 Hz); ^13^C NMR (DMSO) δ: 11.11, 40.77, 45.94, 65.30, 120.13, 129.72, 132.33, 138.45, 141.18, 146.33, 148.45, 161.06; HRMS (ESI, *m*/*z*) Calcd for C_16_H_19_N_6_O_5_S_2_ [M^+^ + H] 439.08529. Found [M^+^ + H] 439.08567. Anal. Calcd for C_16_H_18_N_6_O_5_S_2_: C, 43.82; H, 4.13; N, 19.16. Found: C, 43.55; H, 4.18; N, 19.04.

*N*-(morpholin-1-yl)-4-(3-methyl-5-methylsulfonyl-1*H*-pyrazolo[4,3-*e*][1,2,4]triazyn-1-yl)benzenesulfonamide (**2b**).

Yield 80%. Melting point: 266–269 °C; ^1^H NMR (DMSO) δ: 2.79 (s, 3H), 3.10–3.20 (m, 8H), 3.63 (s, 3H), 3.70–4.00 (m, 4H), 8.15 (d, 2H, J = 8.8 Hz), 8.28 (bs, 1H, exchanged with D_2_O), 8.58 (d, 2H, J = 8.8 Hz); ^13^C NMR (DMSO) δ: 11.12, 36.85, 40.78, 51.24, 55.22, 63.08, 120.19, 128.71, 137.61, 138.29, 140.62, 146.17, 148.34, 161.01; HRMS (ESI, *m*/*z*) Calcd for C_18_H_24_N_7_O_5_S_2_ [M^+^ + H] 482.12748. Found [M^+^ + H] 482.12792. Anal. Calcd for C_18_H_23_N_7_O_5_S_2_: C, 46.43; H, 4.97; N, 21.06. Found: C, 46.08; H, 5.02; N, 20.89.

*N*-(4-methylpiperazin-1-yl)-4-(3-methyl-5-methylsulfonyl-1*H*-pyrazolo[4,3-*e*][1,2,4]triazyn-1-yl)benzenesulfonamide (**2c**).

Yield 60%. Melting point:231–235 °C; ^1^H NMR (DMSO) δ: 1.66–1.69 (m, 4H), 2.79 (s, 3H), 3.19–3.23 (m, 4H), 3.33 (s, 3H), 3.63 (s, 3H), 8.13 (d, 2H, J = 8.8 Hz), 8.59 (d, 2H, J = 8.8 Hz); ^13^C NMR (DMSO) δ: 11.11, 40.74, 41.86, 43.10, 51.49, 120.21, 129.69, 132.21, 138.47, 141.38, 146.41, 148.47, 161.08; HRMS (ESI, *m*/*z*) Calcd for C_17_H_22_N_7_O_4_S_2_ [M^+^ + H] 452.11692. Found [M^+^ + H] 452.11728. Anal. Calcd for C_17_H_21_N_7_O_4_S_2_: C, 45.22; H, 4.68; N, 21.71. Found: C, 44.86; H, 4.74; N, 21.54.

*N*-(1-piperidinyl)-4-(3-methyl-5-methylsulfonyl-1*H*-pyrazolo[4,3-*e*][1,2,4]triazyn-1-yl)benzenesulfonamide (**2d**).

Yield 70%. Melting point: 215–220 °C; ^1^H NMR (DMSO) δ: 1.35–1.41 (m, 2H), 1.53–1.60 (m, 4H), 2.79 (s, 3H), 2.96 (t, 4H, J = 5.2 Hz), 3.63 (s, 3H), 8.05 (d, 2H, J = 8.4 Hz), 8.60 (d, 2H, J = 8.4 Hz); ^13^C NMR (DMSO) δ: 11.11, 22.83, 24.70, 40.77, 46.65, 120.11, 129.45, 133.51, 138.37, 140.87, 146.24, 148.39, 161.03; HRMS (ESI, *m*/*z*) Calcd for C_17_H_21_N_6_O_4_S_2_ [M^+^ + H] 437.10602. Found [M^+^ + H] 437.10638. Anal. Calcd for C_17_H_20_N_6_O_4_S_2_: C, 46.77; H, 4.61; N, 19.25. Found: C, 46.39; 4.67; N, 19.09.

*N*-(1-piperidinyl)-4-(3-methyl-5-piperidine-1*H*-pyrazolo[4,3-*e*][1,2,4]triazyn-1-yl)benzene-sulfonamide (**3d**).

Yield 7%. Melting point: 192–196 °C; ^1^H NMR (CDCl_3_) δ: 1.40–1.44 (m, 2H), 1.60–1.80 (m, 10H), 2.61 (s, 3H), 3.02 (t, 4H, J = 5.6 Hz), 3.98 (t, 4H, J = 5.6 Hz), 7.87 (d, 2H, J =8.8 Hz), 8.55 (d, 2H, J = 8.8 Hz).

#### 4.1.2. Synthesis of Tricyclic Suflonamides (MMs)

Sulfonamide derivative with a methylsulfonyl group (0.33 mmole) was dissolved in anhydrous ethanol (15 mL), and sodium azide (21 mg, 0.33 mmole) was added. The reaction mixture was refluxed until the substrate disappeared (control TLC). Then, the solvent was evaporated and the crude product was purified using column chromatography and CH_2_Cl_2:_ MeOH (50:1) mixture as eluent to give the final compounds as a yellow solid.

*N*-(2-morpholinoeth-1-yl)-4-[7-methyl-5*H*-pyrazolo[4,3-*e*]tetrazolo[1,5-*b*][1,2,4]triazin-5-yl)]benzenesulfonamide (**MM-134**):

Yield 80%. Melting point: 200–202 °C; ^1^H NMR (CDCl_3_) δ: 2.92 (s, 3H), 3.06 (t, 4H, J = 4.8 Hz), 3.77 (t, 4H, J = 4.8 Hz), 7.98 (d, 2H, J = 9.2 Hz), 8.50 (d, 2H, J = 9.2 Hz); HRMS (ESI, *m*/*z*) Calcd for C_15_H_16_N_9_O_3_S [M^+^ + H] 402.10186. Found [M^+^ + H] 402.10189. Anal. Calcd for C_14_H_15_N_9_O_3_S: C, 43.18; H, 3.88; N, 32.37. Found: C, 42.98; H, 3.91; N, 32.22.

*N*-(morpholin-1-yl)-4-[7-methyl-5*H*-pyrazolo[4,3-*e*]tetrazolo[1,5-*b*][1,2,4]triazin-5-yl)]benzenesulfonamide (**MM-136**):

Yield 84%. Melting point: 150–155 °C; ^1^H NMR (CDCl_3_) δ: 2.32 (t, 4H, J = 4.4 Hz), 2.46 (t, 2H, J = 5.6 Hz), 2.91 (s, 3H), 3.09 (t, 2H, J = 5.6 Hz), 3.64 (t, 4H, J = 4.4 Hz), 8.12 (d, 2H, J = 8.8 Hz), 8.46 (d, 2H, J = 8.8 Hz); ^13^C NMR (CDCl_3_) δ: 11.45, 38.92, 52.95, 56.23, 66.73, 119.26, 128.91, 137.83, 140.33, 140.71, 144.37, 145.14, 147.52; HRMS (ESI, *m*/*z*) Calcd for C_17_H_21_N_10_O_3_S [M^+^ + H] 445.15131. Found [M^+^ + H] 445.15107. Anal. Calcd for C_17_H_20_N_10_O_3_S: C, 45.93; H, 4.53; N, 31.51. Found: C, 45.75; H, 4.56; N, 31.38.

*N*-(morpholin-1-yl)-4-[7-methyl-5*H*-pyrazolo[4,3-*e*]tetrazolo[1,5-*b*][1,2,4]triazin-5-yl)]benzenesulfonamide (**MM-137**):

Yield 90%. Melting point: 210–212 °C; ^1^H NMR (CDCl_3_) δ: 2.27 (s, 3H), 2.49 (t, 4H, J = 4.8 Hz), 2.91 (s, 3H), 3.08 (t, 4H, J = 4.8 Hz), 7.94 (d, 2H, J = 8.8 Hz); ^13^C NMR (CDCl_3_) δ: 11.45, 29.68, 45.95, 53.99, 118.92, 129.59, 132.80, 133.43, 140.50, 144.38, 145.20, 147.56; HRMS (ESI, *m*/*z*) Calcd for C_16_H_19_N_10_O_2_S [M^+^ + H] 415.14080. Found [M^+^ + H] 415.14029. Anal. Calcd for C_16_H_18_N_10_O_2_S: C, 46.36; H, 4.37; N, 33.79. Found: C, 46.16; H, 4.40; N, 33.65.

*N*-(morpholin-1-yl)-4-[7-methyl-5*H*-pyrazolo[4,3-*e*]tetrazolo[1,5-*b*][1,2,4]triazin-5-yl)]benzenesulfonamide (**MM-139**):

Yield 86%. Melting point:212–214 °C; ^1^H NMR (CDCl_3_) δ: 1.43 (t, 2H, J = 5.6 Hz), 1.65 (q, 4H, J = 5.6 Hz), 2.91 (s, 3H), 3.02 (t, 4H, J = 5.6 Hz); HRMS (ESI, *m*/*z*) Calcd for C_16_H_18_N_9_O_2_S [M^+^ + H] 400.12991. Found [M^+^ + H] 400.12969. Anal. Calcd for C_16_H_17_N_9_O_2_S: C, 48.11; H, 4.28; N, 31.56. Found: C, 47.89; H, 4.33; N, 31.34.

### 4.2. Biological Studies

#### 4.2.1. Chemicals

Trypsin-EDTA and all culture media (RPMI-1640, DMEM-F12, MEM) were purchased from Biowest (CytoGen, Zgierz, Poland). Acridine orange/ethidium bromide (AO/BE), amino acids solution (MEM), buffered saline (PBS), *β*-mercaptoethanol, penicillin-streptomycin solution stabilized, fetal bovine serum (FBS), dimethyl sulfoxide (DMSO), and MTT 3-(4,5-dimethylthiazol-2-yl)-2,3-diphenyltetrazolium bromide were supplied by Merck/Sigma Aldrich Chemical Co (Burlington, MA, USA). FITC Annexin V Apoptosis Detection Kit I was purchased from B.D. Biosciences (Franklin Lakes, NJ, USA). MitoTracker Red was purchased from Invitrogen (Waltham, MA, USA).

#### 4.2.2. Cell Culture

Cancer cell lines: BxPC-3 (pancreas adenocarcinoma, ATCC^®^ CRL-1687^TM^), HCT-116 (colorectal carcinoma, ATCC^®^ CCL-247™), PC-3 (prostate cancer, ATCC^®^ CRL-1435^TM^), and normal cell lines: L929 (mouse fibroblast, ATCC^®^ CCL-1™) and WI-38 (human lung fibroblasts, ATCC^®^ CCL-75^TM^) were obtained from American Type Culture Collection (ATCC, Rockville, MD, USA). Compositions of culture media used to cultivate cells are presented in Table 8.

Cells were grown at 37 °C in a humidified atmosphere of 5% CO_2_ in the air. The culture medium was changed every 24–48 h. Subculture was performed using 0.25% trypsin/EDTA after cells reached confluence.

#### 4.2.3. MTT Assay

To estimate cell viability following 72 h incubation with the tested MM-compounds (**MM134**, **MM136**, **MM137**, and **MM139**), the MTT test was used according to the PN-EN ISO 10993-5. It is a quantitative method based on the tetrazolium yellow dye MTT [3(4,5-dimethyl-2-thiazolyl)-2,5-diphenyl-2*H*-tetrazolium bromide], which is converted by living cells to a purple product, formazan, the concentration of which was measured colorimetrically. The MTT assay was performed according to the procedure described by Mosmann [87] with modifications. At a density of approximately 8 × 10^3^ cells per 100 µL/well, 96-well plates were seeded. Following the given incubation period in controlled conditions (37 °C; 5% CO_2_), cells were exposed to different concentrations of tested compounds in DMSO (range 0.05–3 µM) in a volume of 100 µL medium per well. Final solvent concentration of DMSO was < 0.5% *v*/*v* [88]. The experimental design included vehicle controls and blanks (wells without cells).

After 72 h of incubation of cells with tested MM-compounds, 20 µL of MTT tetrazolium salt (5 mg/mL in PBS) was added to each well, and plates were incubated once again in a humidified atmosphere for 3 h (37 °C; 5% CO_2_). Following the incubation time, the solutions were removed and 100 µL of DMSO was added to dissolve the formazan complexes. Subsequently, a spectrophotometer (microplate reader Power Wave XS BioTek Instruments, Inc., Winooski, VT, USA) reading was performed at 570 nm. The experiments were performed in duplicates.

### 4.3. Apoptosis Detection

#### 4.3.1. Flow Cytometry Analysis with Annexin V-FITC Staining

The Annexin V-FITC Apoptosis Detection Kit was used to estimate the apoptosis induction following 24 and 48 h incubation with the tested MM-compounds. During apoptosis, phosphatidylserine is transformed from the inner cell membrane to the cell surface, which can be detected by the annexin-FITC complex. The addition of propidium iodide to the reaction mixture allows the assessment of cell membrane integrity. Using flow cytometry analysis, four subpopulations of cells can be distinguished: alive (non-stained), necrotic (propidium iodide stained), early apoptotic (annexin V stained cells), or late-apoptotic cells (propidium iodide and annexin V stained) [89]. BxPC-3 and PC-3 cells were seeded at appropriate density on a 6-well plate (Table 9).

After 24 h, cells were exposed to two concentrations of tested MM-compounds obtained in the 72 h MTT assay: IC_50_, and 2xIC_50_. The experimental design included vehicle control (final solvent concentration was < 0.5% *v*/*v* DMSO), and cells treated with 2 µM SN-38 (active metabolite of irinotecan) used as a positive compensation control. Cells were left for incubation for another 24 or 48 h (37 °C; 5% CO_2_). After exposure, cells were trypsinized and transferred to cytometric tubes, left for 40 min, and centrifuged at 1400 rpm for 10 min at 4 °C. Afterward, the supernatant was removed, and the precipitate was diluted in 1 mL of PBS. The rest of the procedure was performed according to the manufacturer’s instructions. The results were obtained from three independent experiments.

#### 4.3.2. Dual Acridine Orange/Ethidium Bromide (AO/EB) Fluorescent Staining

Acridine orange/ethidium bromide (AO/EB) staining is used to detect nuclear alterations and the development of apoptotic bodies, which are both indicative of apoptosis. Acridine orange penetrates into living cells, emitting green fluorescence after intercalation into DNA. The second dye, ethidium bromide, emits red fluorescence in cells with an altered cell membrane. The cells are divided into three categories as follows: living cells (green nucleus with red orange cytoplasm); apoptotic (green irregularly nuclei with chromatin condensation or fragmentation); and necrotic cells (uniformly orange-stained cell nuclei) [90]. Programmed cell death is measured by counting the number of cells that undergo apoptosis under a fluorescent microscope [46].

BxPC-3 and PC-3 cells were seeded at an appropriate density on 12-well plates (Table 9). After 24 h, cells were exposed to **MM134**, **MM136**, **MM137** and **MM139** in concentrations followed by IC_50_ values obtained in the MTT assay (IC_50_ and 2xIC_50_ concentration) for 24 or 48 h. Following the incubation period, cells were incubated with fluorochromes (AO/EB: 100 µM; 1:1, *v*/*v*) for 5 min at 37 °C in the dark.

Viable, apoptotic, or necrotic cells were distinguished using this method based on differential uptake of fluorescent DNA-binding dyes (AO/EB) with the structural aspect of chromatin condensation in the stained nucleus. Cells were analyzed using a fluorescence microscope (Olympus BX60 F5 Olympus Optical Co., Ltd., Nagano, Japan) at 360 nm. The results were obtained from three independent experiments.

#### 4.3.3. Changes in Transmembrane Mitochondrial Potential-MitoTracker Red (ΔΨm)

Mitotracker Red is deposited in the mitochondrial matrix in response to changes in the inner transmembrane potential of the mitochondria. The intensity of fluorescence reflects the fitness of the mitochondria and alters with changes in MMP [91]. BxPC-3 and PC-3 cells were seeded at appropriate density on a 96-well plates (Table 9).

Cells were incubated with the following concentrations of **MM134**, **MM136**, **MM137**, and **MM139**: 0.5xIC_50_, IC_50_ and 2xIC_50_. After appropriate incubation time, cells were incubated with MitoTracker Red (0.1 µM/200 µL PBS/well) for 40 min. Afterward, MitoTracker Red solution was removed from the wells and PBS (200 µL/well) was added. Fluorescence was read at an absorbance/emission of 581/644 nm using the SpectraMax^®^ i3x Multi-Mode Detection Platform.

### 4.4. Computational Studies

#### 4.4.1. Molecular Docking Simulations

Triazine sulfonamide derivatives were designed and evaluated for their binding interactions with various drug targets involved in cancer pathophysiology with the intent of identifying their probable mechanism of action for their anticancer potential. Three-dimensional structures of all triazine sulfonamide derivatives, i.e., **MM129**, **MM131**, **MM134**, **MM136**, **MM137**, and **MM139**, were prepared by using the ChemDraw Ultra 9.0 tool. These designed ligands were computationally screened against ABL tyrosine-protein kinases (ABL1, ABL2), serine/threonine-protein kinases (AKT1, AKT2, AKT3), breakpoint cluster region protein (BCR), tyrosine-protein kinase (BTK), carbonic anhydrases (CA-IX and CA-XII), cyclin-dependent kinases (CDK2, CDK4, CDK6, and CDK7), intercellular adhesion molecule 1 (ICAM-1), mechanistic target of the rapamycin (mTOR1 and mTOR2) and programmed cell death 1 ligand 1 (PD-L1) by using molecular docking simulation [69].

Three-dimensional structure models of all macromolecular targets having an active involvement in cancer pathophysiology in humans were procured from the protein data bank (PDB) [51].

For the process validation of molecular docking simulation, the structure models of all the prepared macromolecular targets were initially docked against their reference ligands, which were already complex in their structural models. All the reference ligands, i.e., J8N, J92, MHR, and ATP were docked against their respective macromolecular targets, i.e., ABL1, ABL2, AKT1, AKT2, AKT3, BCR, BTK, CA-IX, CA-XII, CDK2, CDK4, CDK6, CDK7, ICAM-1, mTOR, mTOR2 and PD-L1 for the process validation [67].

After successful validation of all the macromolecular targets used in the current study, the designed triazine sulfonamide analogs were screened against them to identify their affinities against each of the macromolecular targets.

#### 4.4.2. Molecular Dynamics Simulations

The obtained docking results were further validated for the stability of the macromolecular drug–receptor complex concerning time by performing molecular dynamics simulation. Based upon the best docking score, the macromolecular complexes of the ligands **MM136**, **MM137**, and **MM139** against AKT1 and PD-L1 enzyme were shortlisted given the best docking results for performing a dynamics simulation at the molecular level for a time frame of 100 nanoseconds (ns) at constant temperature and pressure conditions [69,92].

#### 4.4.3. Drug Likeness and ADMET Prediction

The potential drug-like molecule needs to possess distinct properties that facilitate long-lasting therapeutic effects inside the human body. In the drug development process, it is crucial to estimate the ADME parameters (absorption, distribution, metabolism, and excretion) of a given molecule. Computer models are reliable for the prediction of the pharmacokinetic profile of a newer ligand and may constitute an alternative to biological experiments, leading to a reduction in animal use in drug research, but they cannot fully replace them. Swiss-ADME web tool (http://www.swissadme.ch, accessed on 1 May 2022) [74] was used to estimate critical physicochemical, pharmacological, and drug-like properties of tested compounds based on the Lipiski rule, which states that orally active agents are prohibited from having more than one violation of the following rules: (1) the log P of the octanol/water partition coefficient (log P) should not be higher than five; (2) the molecular weight of the compound should be less than 500 Da; (3) the number of hydrogen bond donors should not be greater than five; and (4) the number of hydrogen donors should not be greater than ten [93,94]. SwissADME allows easy estimation of major pharmacokinetic parameters including, passive human gastrointestinal absorption (HIA) and blood–brain barrier (BBB) permeation [95]. SwissADME allows screening compounds for their cytochrome P450 (CYP) inhibitory activity. This is an important issue given that CYP isoforms are involved in drug elimination through metabolic biotransformation and thus impact the efficiency of therapy and affect the drug toxicity and adverse effects [93].

## 5. Conclusions

The conducted research provides the necessary information on the biological response of cancer and normal human cells to MM-compounds with an innovative, modified chemical structure, showing oncotherapeutic potential. Pyrazolo[4,3-*e*]tetrazolo[1,5-*b*][1,2,4]triazine sulfonamides are emerging as an important scaffold for drug discovery. Here we have shown the utility of four pyrazolo-triazine derivatives (**MM134**, **MM136**, **MM137,** and **MM139**) as potential anticancer compounds with selective cytotoxic and proapoptotic properties in various cancer cell line models. Our data indicate that antitumor potential may result from the dual inhibition of AKT-mTOR and PD-1-PD-L1 pathways in cancer cells. The exploration of biological activity of other analogs of MM derivatives may lead to optimization and discovery of novel, safe and potent inhibitors of these pathways with more selective action against neoplastic cells and minimized cytotoxicity against normal human cells.

## Figures and Tables

**Table 1:** Mean IC_50_ obtained after a 72 h incubation of tested cells with MM-compounds in MTT assay ± SD.

MM-Compounds	Cell Lines				
	Mean IC_50_ [µM]				
	BxPC-3	HCT-116	PC-3	L929	WI-38
**MM134**	0.32 ± 0.1	0.38 ± 0.03	0.16 ± 0.02	0.58 ± 0.005	0.65 ± 0.07
**MM136**	0.25 ± 0.08	0.25 ± 0.07	0.13 ± 0.01	0.22 ± 0.08	0.48 ± 0.09
**MM137**	0.16 ± 0.04	0.14 ± 0.02	0.11 ± 0.007	0.18 ± 0.0008	0.27 ± 0.04
**MM139**	0.33 ± 0.14	0.35 ± 0.05	0.17 ± 0.003	0.26 ± 0.02	0.54 ± 0.06

**Table 2:** Results of MitoTracker Red analysis in tested cancer cell lines. Data for individual compounds are presented as mean percentage of control group fluorescence intensity ± SD value.

BxPC-3			
24 h incubation			
Compound/Concentration	0.5IC_50_	IC_50_	2xIC_50_
**MM134**	88.19 ± 13.62	81.23 ± 11.94	78.31 ± 14.34
**MM136**	82.83 ± 13.02	65.68 ± 4.58	57.67 ± 5.56
**MM137**	89.34 ± 5.91	79.5 ± 3.34	79.22 ± 2.22
**MM139**	81.28 ± 14.44	66.73 ± 3.72	64.23 ± 6.08
48 h incubation			
**MM134**	79.98 ± 11.47	66.04 ± 16.7	68.95 ± 7.78
**MM136**	69.08 ± 4.40	62.37 ± 0.05	61.83 ± 0.46
**MM137**	84.62 ± 9.01	79.8 ± 6.76	73.8 ± 2.58
**MM139**	76.9 ± 6.40	60.35 ± 0.91	65.95 ± 12.53
**PC-3**			
24 h incubation			
**MM134**	100.35 ± 1.57	82.5 ± 2.21	82.34 ± 2.13
**MM136**	96.3 ± 5.74	92.78 ± 0.9	86.9 ± 7.22
**MM137**	96.26 ± 2.83	87.9 ± 2.71	82.5 ± 7.99
**MM139**	89.2 ± 6.28	87.28 ± 15.8	78.14 ± 6.55
48 h incubation			
**MM134**	82.1 ± 14.45	80.26 ± 13.44	64.56 ± 3.33
**MM136**	81.34 ± 0.4	61.24 ± 3.62	47.38 ± 2.74
**MM137**	78.26 ± 2.62	75.49 ± 5.55	64.19 ± 8.72
**MM139**	78.91 ± 15.21	65.93 ± 19.56	61.28 ± 9.16

**Table 3:** The coordinates of the grid box for all the anticancer targets used in the current study.

Drug Target	PDB Code	x-D	y-D	z-D	Spacing (Ả)	x Center	y Center	z Center
ABL1	4wa9	40	40	40	0.431	23.95	127.557	14.335
ABL2	2xyn	40	40	40	0.536	−54.025	48.668	−7.666
AKT1	3mvh	40	40	40	0.453	17.948	−1.885	27.56
AKT2	3d0e	40	40	40	0.475	22.521	−19.611	7.41
AKT3	2x18	40	40	40	0.458	25.066	66.608	−19.071
BCR	5n7e	40	40	40	0.636	22.531	−2.925	111.276
BTK	3gen	40	40	40	0.492	−16.796	6.794	−14.126
CA-IX	6qn5	40	40	40	0.375	−29.939	−3.656	0.027
CA-XII	6qnl	40	40	40	0.375	−41.046	6.288	31.866
CDK2	3bhu	40	40	40	0.375	−7.638	20.962	−21.4
CDK4	2w9z	50	50	50	0.453	20.281	25.506	8.713
CDK6	5l2s	50	50	50	0.403	22.452	38.566	−8.687
CDK7	1ua2	40	40	40	0.375	41.304	−4.892	23.033
ICAM-1	5mza	40	40	40	0.531	6.393	−16.531	9.728
mTOR1	6bcx	40	40	40	0.625	247.93	204.864	264.981
mTOR2	6zwo	40	40	40	0.625	249.818	191.487	206.7
PD-L1	7bea	40	40	40	0.492	−5.537	−10.417	19.104

**Table 4:** Molecular docking results of six triazine sulfonamide analogs as well as all the reference ligands.

S. No.	Ligand	Binding Energy ABL1 (4wa9)	Binding Energy ABL2 (2xyn)	Binding Energy AKT1 (3mvh)	Binding Energy AKT2 (3d0e)	Binding Energy AKT3 (2x18)	Binding Energy BCR (5n7e)	Binding Energy BTK (3gen)	Binding Energy CA-IX (6qn5)	Binding Energy CA-XII (6qnl)	Binding Energy CDK2 (3bhu)	Binding Energy CDK4 (2w9z)	Binding Energy CDK6 (5ls2)	Binding Energy CDK7 (1ua2)	Binding Energy ICAM-1 (5mza)	Binding Energy mTOR1 (6bcx)	Binding Energy mTOR2 (6zwo)	Binding Energy PD-L1 (7bea)
1	Reference Ligand	−9.35 (AXI)	−9.28 (VX6)	−10.51 (WFE)	−9.54 (G93)	−4.4 (EPE)	-	−9.31 (B43)	−8.18 (J8N)	−7.63 (J92)	−7.67 (MHR)	−5.7 (NAG)	−9.4 (6ZV)	−7.64 (ATP)	-	−3.26 (ATP)	−4.71 (AGS)	−10.09 (TK2)
3	**MM129**	−9.71	−9.17	−10.70	−9.52	−6.68	−8.63	−8.52	−8.44	−8.21	−9.61	−8.97	−10.83	−10.04	−6.86	−8.31	−8.12	−9.86
4	**MM131**	−9.48	−9.23	−9.92	−9.62	−6.74	−8.62	−8.76	−7.90	−7.87	−8.80	−8.52	−9.01	−8.74	−6.51	−7.19	−7.44	−9.88
5	**MM134**	−9.88	−10.08	−11.35	−11.81	−6.94	−9.51	−10.11	−8.01	−8.04	−9.63	−8.08	−9.69	−9.14	−7.20	−8.45	−7.64	−10.30
6	**MM136**	−9.53	−9.81	−12.42	−9.93	−6.63	−9.04	−9.55	−8.39	−8.54	−8.60	−8.97	−9.26	−9.40	−7.68	−9.08	−8.79	−12.33
7	**MM137**	−10.11	−10.46	−11.51	−10.79	−6.84	−8.49	−10.06	−7.86	−8.52	−8.31	−8.78	−8.31	−9.78	−7.19	−8.57	−7.95	−11.54
8	**MM139**	−10.42	−9.84	−12.16	−10.55	−7.24	−8.52	−10.51	−9.34	−9.06	−10.02	−9.62	−9.68	−9.88	−7.68	−8.46	−8.29	−11.43

**Table 5:** Chosen physicochemical properties of tested compounds.

Property	Parameter	MM129	MM131	MM134	MM136	MM137	MM139
Flexibility	Num. rotatable bonds	5	5	6	3	3	3
Lipophilicity	XLOGP3	−0.31	−0.21	−0.30	−0.15	0.04	1.07
Molecular weight	MW	459.44 g/mol	389.39 g/mol	444.47 g/mol	401.40 g/mol	414.44 g/mol	399.43 g/mol
Polarity	TPSA	178.97 Å^2^	161.46 Å^2^	153.70 Å^2^	141.67 Å^2^	135.68 Å^2^	132.44 Å^2^
Saturation	Fraction Csp3	0.35	0.29	0.41	0.33	0.38	0.38

**Table 6:** Predicted ADMET properties of tested compounds.

Compound	MM129	MM131	MM134	MM136	MM137	MM139
GI absorption	Low	Low	Low	Low	High	High
BBB permeant	No	No	No	No	No	No
P-gp substrate	Yes	Yes	Yes	Yes	Yes	Yes
CYP2D6 Inhibitor	No	No	No	No	No	No
CYP3A4 Inhibitor	No	No	Yes	No	No	No

**Table 7:** Predicted molecular properties related to Lipinski’s rule of five.

Compound	MM129	MM131	MM134	MM136	MM137	MM139
Molecular weight	459.44 g/mol	389.39 g/mol	444.47 g/mol	401.40 g/mol	414.44 g/mol	399.43 g/mol
Num. H-bond acceptors	12	10	11	10	10	9
Num. H-bond donors	1	2	1	0	0	0
Consensus Log *P*_o/w_	0.07	0.28	0.35	0.51	0.41	1.31
Lipinski violation	1	1	1	1	1	1

**Table 8:** Compositions of culture media used to cultivate cells.

Type of Cells	Medium	Supplements
BxPC-3 HCT-116	RPMI-1640	10% (*v*/*v*) FBS, L-Glutamine, 25 mM Hepes, 1% penicillin-streptomycin
PC-3	DMEM-F12	10% (*v*/*v*) FBS, L-Glutamine, 15 mM Hepes 1% penicillin-streptomycin
L929	RPMI-1640	10% (*v*/*v*) FBS, L-Glutamine, 25 mM Hepes, 1% penicillin-streptomycin 1% β-mercaptoethanol
WI-38	MEM	10% (*v*/*v*) FBS, L-Glutamine, 25 mM Hepes 1% penicillin-streptomycin

**Table 9:** Density of BxPC-3 and PC-3 cells used in the experimental series in Annexin V-FITC staining (the white panel), MitoTracker Red (the yellow panel) and dual acridine orange/ethidium bromide fluorescent staining (the grey panel).

Cancer Cell Lines	Cell Density/2 mL Culture Medium Annexin V-FITC	
	24 h Incubation	48 h Incubation
BxPC-3	6 × 10^5^	5 × 10^5^
PC-3	7 × 10^5^	5 × 10^5^
	**Cell density/200 µL culture medium** **MitoTracker Red**	
	24 h incubation	48 h incubation
BxPC-3	2 × 10^4^	1.5 × 10^4^
PC-3	1.2 × 10^4^	1.5 × 10^4^
	**Cell density/mL culture medium** **AO/EB**	
	24 h incubation	48 h incubation
BxPC-3	1.5 × 10^5^	1 × 10^5^
PC-3	1.5 × 10^5^	1 × 10^5^

## Data Availability

The data presented in this study are available in the main text of this article/supplementary materials of this article or on request from the corresponding author.

## Supplementary Materials

The following supporting information can be downloaded at: https://www.mdpi.com/article/10.3390/ijms23115892/s1.

## Abbreviations

ABL	ABL tyrosine-protein kinase
AIF	apoptosis-inducing factor
AKT	serine/threonine-protein kinase
AO/EB	Acridine orange/ethidium bromide
BBB	blood–brain barrier
BTK	Bruton’s tyrosine kinase
CA	carbonic anhydrase
CDK	cyclin-dependent kinases
Csp3	carbons in the sp3 hybridization
CYP	cytochrome P450
DMSO	dimethyl sulfoxide
FBS	fetal bovine serum
HIA	human gastrointestinal absorption
ICAM-1	intercellular adhesion molecule 1
MEM	amino acids solution
MMP	mitochondria membrane potential
mTOR	mechanistic target of the rapamycin
MTT	3-(4,5-dimethylthiazol-2-yl)-2,3-diphenyltetrazolium bromide
MW	molecular weight
PBS	buffered saline
PD-1	programmed death receptor-1
PDB	protein data bank
PD-L1	programmed death ligand-1
PGP	glycoprotein P
PI3K	phosphoinositide-3-kinase
PTEN	phosphatase and tensin homolog
RMSD	root mean square deviation
RMSF	root mean square fluctuation
TP53	cellular tumor antigen p53
TPSA	topological polar surface area
