# Supplementary material for: Preparation of Novel Pyrazolo[4,3-e]tetrazolo[1,5-b][1,2,4]triazine Sulfonamides and Their Experimental and Computational Biological Studies
Source: Int J Mol Sci. 2022 May 24;23(11):5892. doi: 10.3390/ijms23115892 (PMC9180621; doi:10.3390/ijms23115892)
Supplement: Supplementary file 1 [file ijms-23-05892-s001.zip › SM1.pdf]

A)

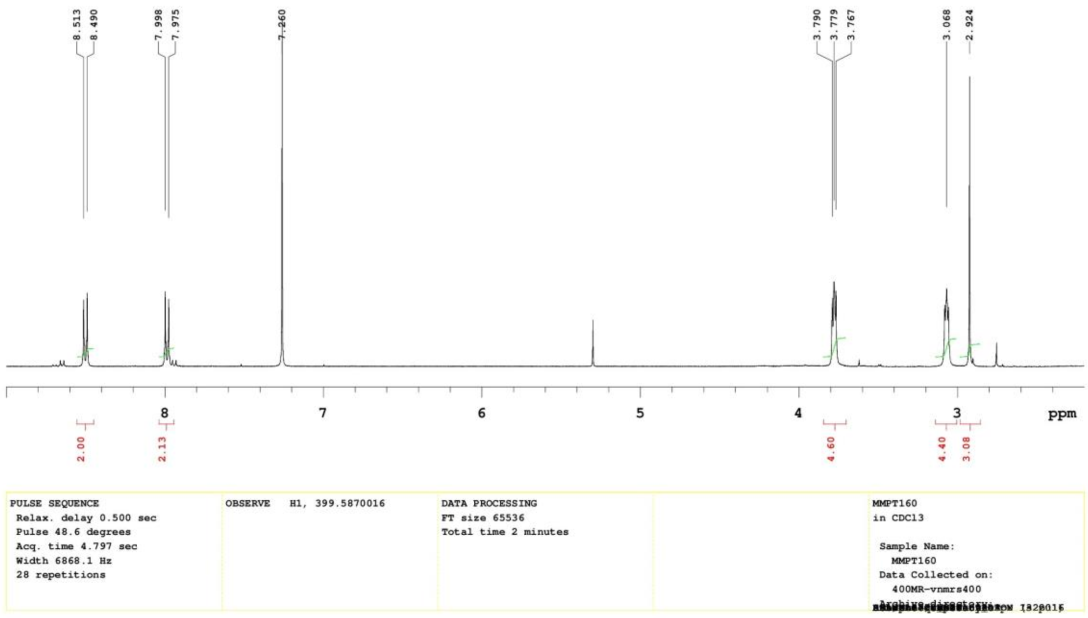
8.513
8.490
7.998
7.975
7.260
3.790
3.779
3.767
3.068
2.924
8
7
6
5
4
3
ppm
2.00
2.13
4.60
4.40
3.08
PULSE SEQUENCE
Relax. delay 0.500 sec
Pulse 48.6 degrees
Acq. time 4.797 sec
Width 6868.1 Hz
28 repetitions
OBSERVE H1, 399.5870016
DATA PROCESSING
FT size 65536
Total time 2 minutes
MMPT160
in CDCl3
Sample Name:
MMPT160
Data Collected on:
400MR-vnmrs400

B)

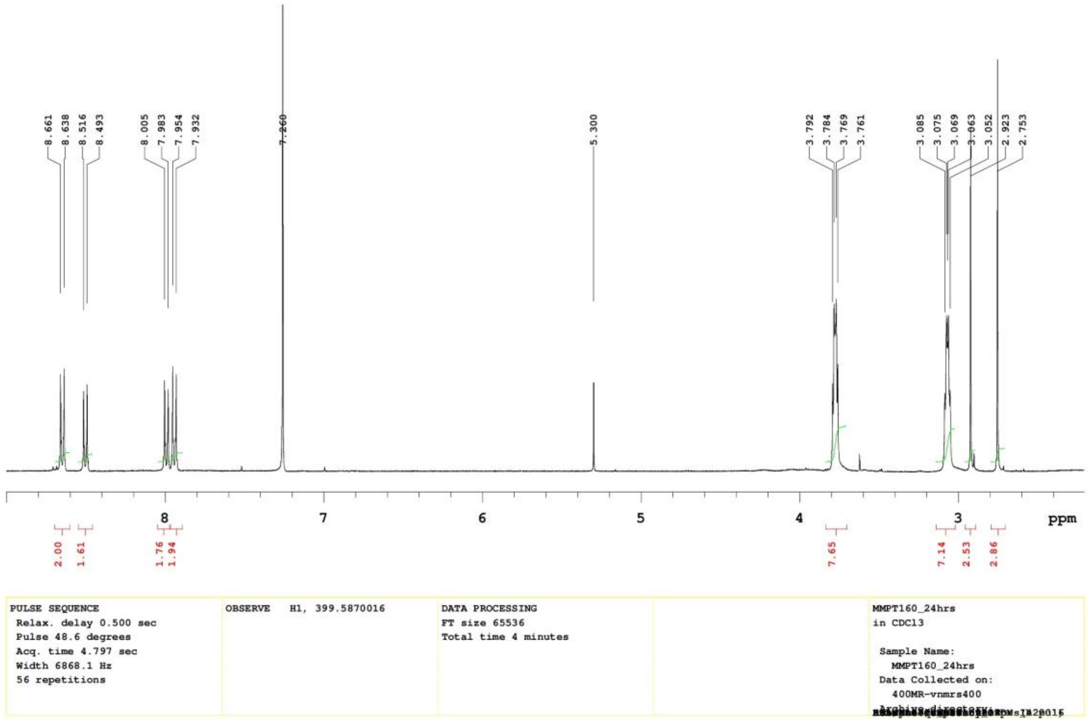
8.661
8.638
8.516
8.493
8.005
7.983
7.954
7.932
7.260
5.300
3.792
3.784
3.769
3.761
3.085
3.075
3.069
3.063
3.052
2.923
2.753
8
7
6
5
4
3
ppm
2.00
1.61
1.76
1.94
7.65
7.14
2.53
2.86
PULSE SEQUENCE
Relax. delay 0.500 sec
Pulse 48.6 degrees
Acq. time 4.797 sec
Width 6868.1 Hz
56 repetitions
OBSERVE H1, 399.5870016
DATA PROCESSING
FT size 65536
Total time 4 minutes
MMPT160_24hrs
in CDCl3
Sample Name:
MMPT160_24hrs
Data Collected on:
400MR-vnmrs400

C)

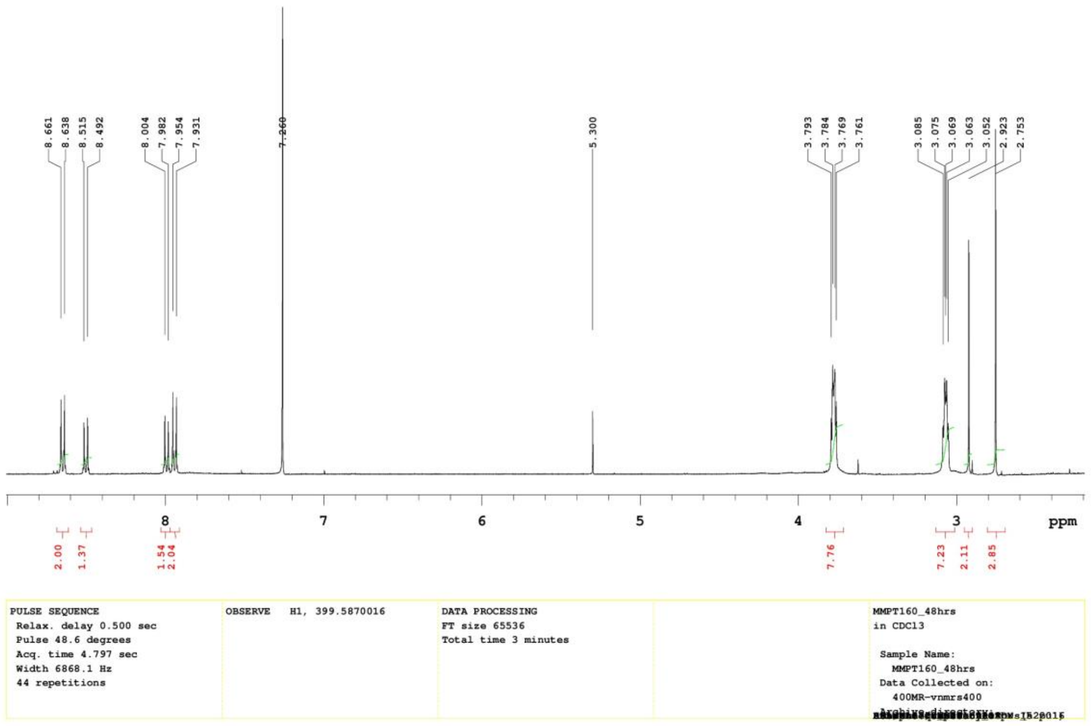

Supplementary material Figure S1: The ^{1}H NMR spectrum (**A**) recorded immediately after solution of the compound **MM134** in deuterated chloroform and repeated (**B**) after 24 h and (**C**) after 48 h.

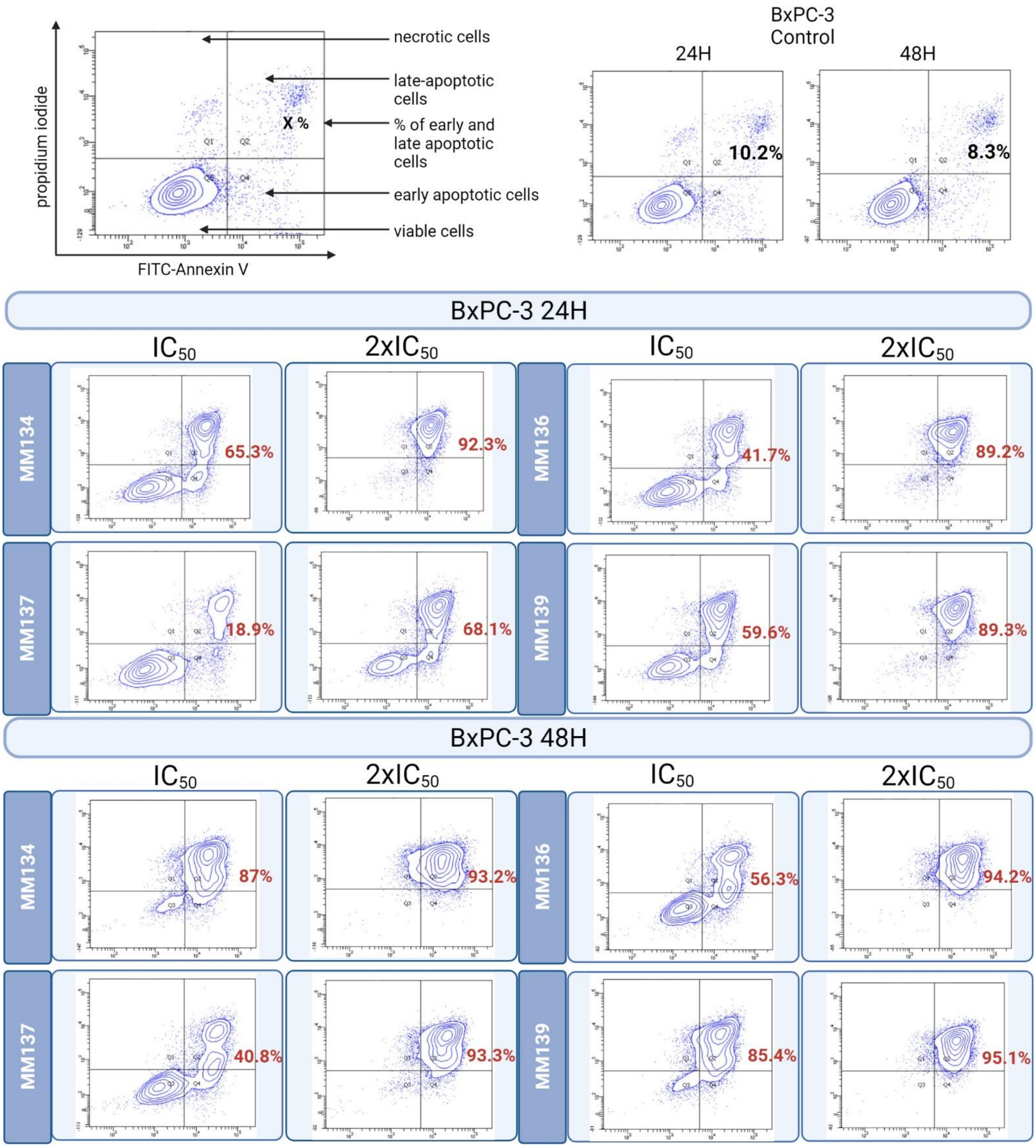

Supplementary material Figure S2: Apoptosis detection by flow cytometry with Annexin V-FITC staining in BxPC-3 cells exposed to IC_{50} and $2xIC_{50}$ concentrations of **MM134**, **-6**, **-7**, and **-9** for 24 and 48-h Left bottom square represents live cells, right bottom early apoptotic cells, the top left square necrotic cells, and top right late apoptotic cells.

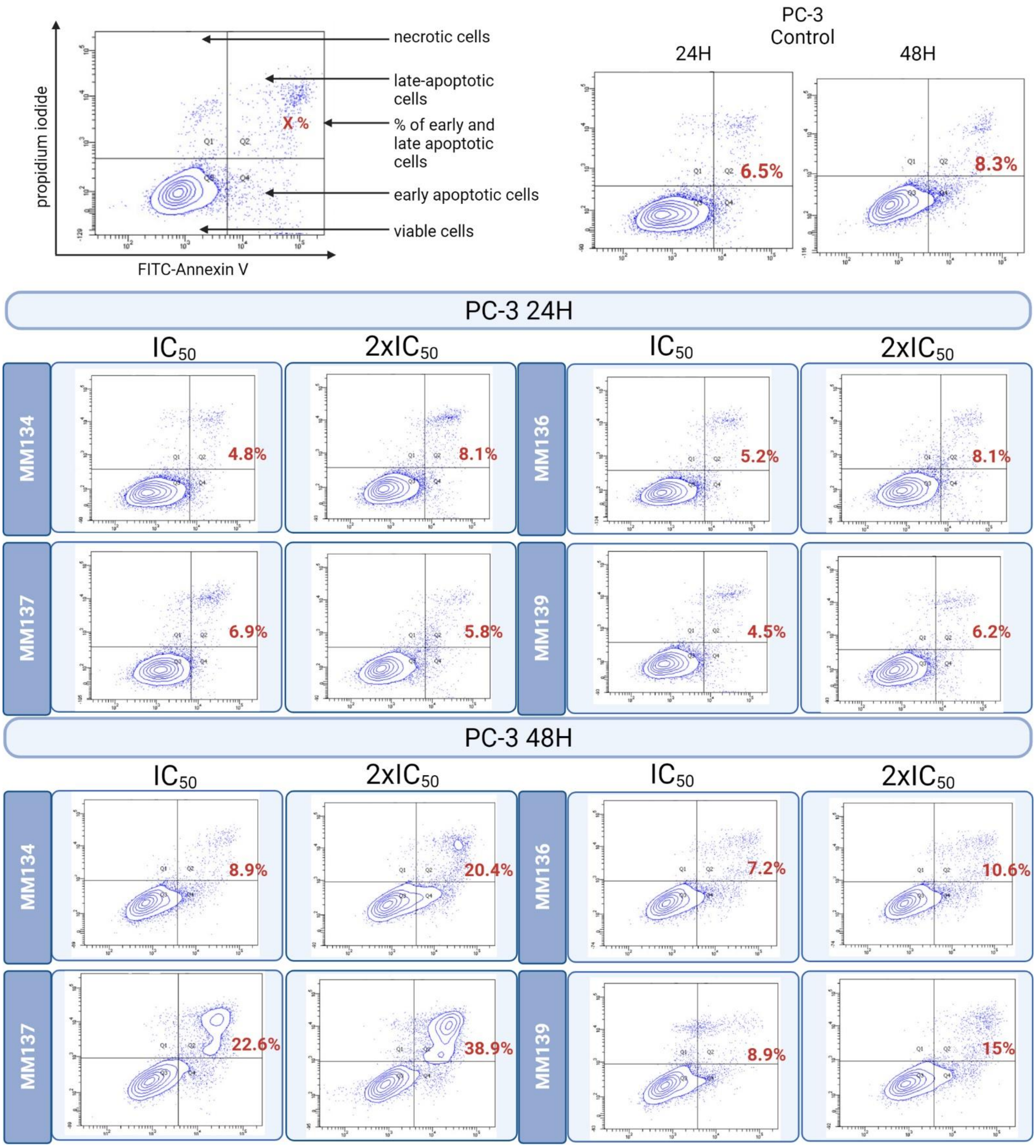

Supplementary material Figure S3: Apoptosis detection by flow cytometry with Annexin V-FITC staining in PC-3 cells exposed to IC_{50} and $2xIC_{50}$ concentrations of **MM134**, **-6**, **-7**, and **-9** for 24 and 48-h Left bottom square represents live cells, right bottom early apoptotic cells, the top left square necrotic cells, and top right late apoptotic cells.
